# Unraveling Assemblage, Functions and Stability of the Gut Microbiota of *Blattella germanica* by Antibiotic Treatment

**DOI:** 10.3389/fmicb.2020.00487

**Published:** 2020-03-25

**Authors:** Rebeca Domínguez-Santos, Ana Elena Pérez-Cobas, Alejandro Artacho, José A. Castro, Irene Talón, Andrés Moya, Carlos García-Ferris, Amparo Latorre

**Affiliations:** ^1^Institute for Integrative Systems Biology, University of Valencia and CSIC, Valencia, Spain; ^2^Institut Pasteur, Biologie des Bactéries Intracellulaires, Paris, France; ^3^CNRS UMR 3525, Paris, France; ^4^Genomics and Health Area, Foundation for the Promotion of Sanitary and Biomedical Research, Valencia, Spain; ^5^Unit of Genetics, Department of Biology, University of the Balearic Islands, Palma, Spain; ^6^Department of Biochemistry and Molecular Biology, University of Valencia, Valencia, Spain

**Keywords:** *Blattella germanica*, endosymbiosis, gut microbiota, antibiotics, functional resilience

## Abstract

Symbiosis between prokaryotes and eukaryotes is a widespread phenomenon that has contributed to the evolution of eukaryotes. In cockroaches, two types of symbionts coexist: an endosymbiont in the fat body (*Blattabacterium*), and a rich gut microbiota. The transmission mode of *Blattabacterium* is vertical, while the gut microbiota of a new generation is mainly formed by bacterial species present in feces. We have carried out a metagenomic analysis of *Blattella germanica* populations, treated and non-treated with two antibiotics (vancomycin and ampicillin) over two generations to (1) determine the core of bacterial communities and potential functions of the gut microbiota and (2) to gain insights into the mechanisms of resistance and resilience of the gut microbiota. Our results indicate that the composition and functions of the bacteria were affected by treatment, more severely in the case of vancomycin. Further results demonstrated that in an untreated second-generation population that comes from antibiotic-treated first-generation, the microbiota is not yet stabilized at nymphal stages but can fully recover in adults when feces of a control population were added to the diet. This signifies the existence of a stable core in either composition and functions in lab-reared populations. The high microbiota diversity as well as the observed functional redundancy point toward the microbiota of cockroach hindguts as a robust ecosystem that can recover from perturbations, with recovery being faster when feces are added to the diet.

## Introduction

Many insect species, like other animals, need microorganisms to help perform essential host functions. One type are endosymbionts that have established a one-to-one (or one-to-few) relationship with the hosts, living intracellularly in specialized host cells, and functionally complementing the host in many ways (reviewed in Latorre and Manzano-Marín, 2017). Another type are ectosymbionts, normally establishing a many-to-one relationship between them and the host, and living in different host organs constituting what is known as the microbiota. The microbiota located in the intestine of many animals is of particular importance, as it contributes to the absorption of nutrients and may perform other functions that help the health status of the host (Dillon and Dillon, 2004; Engel and Moran, 2013; Otani et al., 2014; Douglas, 2015; Emery et al., 2017; Foster et al., 2017; Kwong et al., 2017; Moran et al., 2019). Numerous studies have aimed to characterize the diversity of the gut microbial communities in insects such as termites (Brune, 2014; Brune and Dietrich, 2015), cockroaches (Carrasco et al., 2014; Pérez-Cobas et al., 2015; Pietri et al., 2018), crickets (Santo Domingo et al., 1998), beetles (Vogel et al., 2017; Shukla et al., 2018), bees (Kwong and Moran, 2016), lepidopterans (Broderick et al., 2004; Robinson et al., 2010; Pinto-Tomás et al., 2011), and *Drosophila* (Broderick and Lemaitre, 2012). However, our knowledge is currently limited on the functions of the gut microbiota in insects, as few works have used metagenomics or metatranscriptomics in their studies.

Cockroaches (Blattodea) are a particularly intriguing case since both, endosymbiont and microbiota, coexist in each individual host. On one hand, the obligate endosymbiont *Blattabacterium* is present in special bacterial cells (bacteriocytes) in the fat body, that plays an essential role in nitrogen metabolism (López-Sánchez et al., 2009). On the other hand, the cockroaches contain a rich and diverse microbiota in their hindgut, whose function is not yet well study and understood (Schauer et al., 2012, 2014; Carrasco et al., 2014; Pérez-Cobas et al., 2015; Tegtmeier et al., 2015; Tinker and Ottesen, 2016; Kakumanu et al., 2018; Rosas et al., 2018).

In gregarious insects such as cockroaches, coprophagy is an important way of shaping the gut microbiota, either from the intake of feces from the environment (i.e., horizontal transmission) or transmitted vertically through the eggs or the offspring (reviewed in Onchuru et al., 2018). Not only is transmission important, but also the way in which the microbiota is established in the ecological succession (Nalepa, 1990; Carrasco et al., 2014; Duguma et al., 2015; Johnston and Rolff, 2015). A recent study found that the colonization success of the obligate anaerobic *Fusobacterium* strain FuSL in the gnotobiotic cockroach *Shelfordella lateralis* is dependent on the presence of other microbial species, indicating that the order in which species colonize the gut could determine community structure (Tegtmeier et al., 2015). Also, a study of germ-free *S. lateralis* colonized with different species’ combinations showed that gut environment preferentially selects for lineages that are specifically adapted to it (Mikaelyan et al., 2016). In the case of the German cockroach *Blattella germanica*, the endosymbiont *Blattabacterium* is transmitted vertically from mothers to oocytes as it is the only bacterium present in the eggs (Carrasco et al., 2014). In terms of microbiota transmission, a recent study using the broad-spectrum antibiotic rifampicin to disturb the gut microbiota in one generation demonstrated that bacterial species present in the diet, and particularly in the feces, contribute significantly to gut microbiota acquisition in the next generation (Rosas et al., 2018). However, we are lacking studies on the acquisition of the microbiota until its establishment and stability at adult stage as well as its functional role.

In the present work, we have carried out a metagenomic study of the gut microbiota of *B. germanica* treated and untreated in parallel with two different antibiotics during two consecutive generations in different developmental stages. The main goals of this study were to elucidate the existence of a core of bacterial taxa and associated functions in the gut microbiota of *B. germanica*, as well as to test the changes of the gut microbes during antibiotic treatments and resilience after its cessation.

## Materials and Methods

### *Blattella germanica* Population

*B. germanica* originating from a laboratory population at the Institute of Evolutionary Biology (CSIC-UPF, Barcelona) was reared in climatic chambers at the Cavanilles Institute of Biodiversity and Evolutionary Biology (University of Valencia) in plastic jars with aeration at 26 °C, 65% humidity and 12L:12D photoperiod. Cockroaches were fed dog-food pellets (Teklad, Madison, United States; global 21% protein dog diet, 2021C) and water was supplied *ad libitum.* The antibiotics vancomycin (Alfa Aesar, Germany) and ampicillin (Sigma-Aldrich, Missouri, United States) were supplied with the water at 0.02% (w/v) in two different experiments (named Vancomycin and Ampicillin experiments, respectively). Vancomycin is a glycopeptide antibiotic, acting against Gram-positive bacteria by binding to D-alanyl-D-alanine portion of the bacterial cell wall precursors, and prevents binding of this portion with the PBP (penicillin-binding protein). Ampicillin is a beta-lactam antibiotic acting against Gram-positive and some Gram-negative bacteria, which binds to PBPs, inactivating them and interfering with the cross-linkage of peptidoglycan chain.

### Experimental Design

**FIGURE 1:**
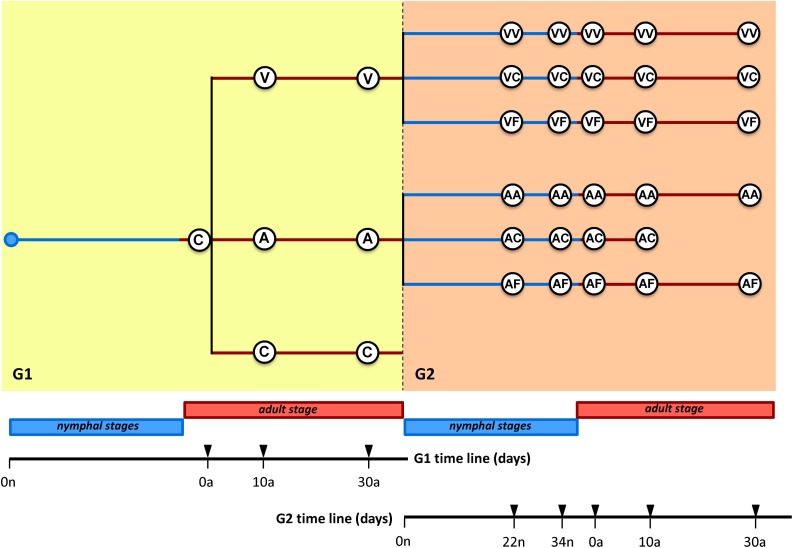
Experimental design. The antibiotics vancomycin (V) and ampicillin (A) were applied separately to synchronized *B. germanica* adults, and two different generations (G1 and G2) were studied. At G1, three populations were performed: without any antibiotic (control C), with vancomycin (V) and with ampicillin (A). At G2, three populations were performed for each antibiotic: with antibiotic (VV or AA), antibiotic-free (VC or AC), or antibiotic-free with feces added to the diet (VF or AF). The numbers on the timelines indicate the time (in days) of nymphal stage (n, blue line) or adult stage (a, orange line) at which female dissections were made. Fat body and hindgut of each sample were collected for DNA analyses (see text and Supplementary Table S1 for additional information).

We started with a synchronized adult population, composed of individuals collected between 0–48 h after adult ecdysis (generation 1, G1) (see Figure 1 and Supplementary Table S1 for a summary of the whole experiment). Before treatment, four female cockroaches were taken from the population and dissected at 0 days (C_0_). Then, the population was divided into three subpopulations: One was never treated with antibiotics and was used as control (C population), and the other two were treated with vancomycin (V population) or ampicillin (A population). Samples were taken at 10 and 30 days in the three G1 populations. When the ootheca were fully formed, adults from each antibiotic population were divided into two groups, with or without antibiotic, to generate the second generation (G2) populations. Newly hatched nymphs’ population from the groups maintained on antibiotic initiated the antibiotic treatment populations (VV and AA populations). The nymphs hatched in antibiotic-free environment were immediately divided into two populations, one without any further treatment giving rise to the VC and AC populations, and the other one supplemented with feces obtained from a control population never treated with antibiotics, yielding the VF and AF populations. At G2, we collected samples at 22 and 34 days that correspond approximately to the nymphal stages n3 and n4 and at 0, 10, and 30 days after ecdysis. The AC30a sampling time point was not taken because none of the individuals reached this age. Overall, 113 samples were analyzed, 52 in the vancomycin experiment (7 and 45 in G1 and G2, respectively), 49 in the ampicillin experiment (7 and 42 in G1 and G2, respectively) and 12 in control. We analyzed the microbiota composition and functions in the hindgut by metagenomics, and quantified the *Blattabacterium* population in the fat body by qPCR.

### Cockroach Dissection

Cockroaches were anesthetized by CO_2_, and dissected under stereomicroscope. The fat body was recovered in Ringer’s solution. The hindgut was opened and cleaned with Ringer’s solution. Both tissues were frozen in liquid nitrogen and stored at −80°C until DNA extraction.

### DNA Extraction and Sequencing

Hindgut and fat body were ground with a sterile plastic pestle. Hindgut total DNA was obtained using the JetFlex Genomic DNA Extraction kit (Genomed, Germany) following the manufacturer’s recommendations, adding lysozyme (20 mg/ml) to the cell lysis buffer to break Gram-positive bacterial cell wall, and used for metagenomic sequencing using the Illumina MiSeq (2 × 300 bp) technology at the FISABIO (Valencia, Spain). Total DNA from the fat body was extracted following the protocol described in Llop et al. (1999) with an additional phenolization and was used for qPCR analyses. DNA was quantified with Qubit (Thermo Fisher, Massachusetts, United States).

### Bioinformatics Methods: Quality Control, Taxonomic and Functional Analysis

From the raw sequencing data, the adaptors were removed by using Cutadapt (Martin, 2011). To remove short reads, reads with low mean quality, reads with high percentage of ambiguous bases and to trim the 3′ ends with low quality the PRINSEQ (Schmieder and Edwards, 2011) was used. The FLASH software was used to join overlapping pairs in order to obtain longer sequences (Magoč and Salzberg, 2011). To discard host genome reads from the metagenomes, Bowtie 2 software was used (Langmead and Salzberg, 2012). Negative control samples were included and sequencing produced reads whose number was negligible.

Taxonomic assignments of the metagenomes were carried out through Kaiju and the non-redundant bacteria database (Menzel et al., 2016), a program for computationally efficient and sensitive taxonomic classification of high-throughput sequencing reads from metagenomic sequencing experiments.

To assign functional annotations, the reads were previously assembled into contigs via Ray (v2.3.1) (Boisvert et al., 2012). Then, we used Prodigal (v2.6.3) (Hyatt et al., 2010) to identify genes inside contigs and HMMER (v3.1b2) (Durbin et al., 1998) against the prokaryotic models TIGRFAM database (v15.0) (Selengut et al., 2007). The abundance of the annotated genes was finally measured by counting aligned reads to them via megaBLAST (v2.2.26) (Altschul et al., 1990), at 97% of identity over 100% query coverage. Functional characterization of antibiotic resistant genes was performed by aligning our reads against Antibiotic Resistance Genes Database (ARDB) (Liu and Pop, 2009).

### Biostatistics Methods

Taxonomic and functional annotations provide bacterial species and gene compositional abundance matrices, respectively. The taxonomy was based on the non-redundant bacteria database and the functional annotation on the TIGRFAMs protein family database. We used these abundance tables to carry out comparative analyses. The analyses were performed at different classification levels: family in the case of taxonomy and gene and biological process (main role and sub role levels) in the case of functions. The analyses were performed with in-house R scripts (R version: v3.1) (R Core Team, 2014).

Alpha diversity (estimated at the bacterial species level) of the different sample points was based on the Shannon and Chao1 indexes and statistically tested with the Wilcoxon signed-rank test implemented in the R library vegan (Oksanen et al., 2017). We used canonical correspondence analysis (CCA) to plot and compare groups of samples on two-dimensional maps. The analysis was based on the Bray-Curtis dissimilarity metric. The CCAs were analyzed by the multivariate analysis of variance (beta diversity) and statistically tested by means of the Adonis test also implemented in the vegan R package.

Univariate comparative analyses were performed (visualized in heatmaps) to identify both taxonomic and functional shifts between groups of samples. To determine shifts, each feature (taxon, function or sub role) was tested for exhibiting statistically significant differences between two groups according to Wilcoxon test. Moreover, in order to reduce false positives, a false discovery rate (FDR) adjusted *p*-value below 0.05 was used and 90% confidence intervals for the relative abundances of the two groups could not overlap.

### Quantitative PCR

To check if the antibiotics have an effect on the endosymbiont and therefore in the host–microbiota interaction, the *Blattabacterium* population was analyzed by qPCR using ArialMx Real-Time PCR System (Agilent Technologies, Germany). The genes *ure*C (accession number NC_013454.1) and *actin*5C (accession number AJ861721.1) were used as specific of the endosymbiont population and the host control, respectively. The primers UC1F: 5′-GTCCAGCAACTGGAACTATAGCCA-3′ and UC1R: 5′-CCTCCTGCACCTGCTTCTATTTGT-3′ were used for the *ure*C gene, and ActinF: 5′-CACATACAACTCCATTATGAAGTGCGA-3′ and ActinR: 5′-TGTCGGCAATTCCAGGGTACATG-3′ were used for the *actin*5C gene, as previously described (Rosas et al., 2018). Statistical differences among samples of qPCR results were evaluated applying the Wilcoxon test (adjusted *p*-values by FDR method).

## Results

A total of 113 individuals corresponding to 36 time points of the vancomycin and ampicillin experiments were analyzed by metagenomic sequencing (Figure 1 and Supplementary Table S1). The average number of reads per sample was 743,008 (484,377 after quality filtering/host sequences decontamination) and a mean of 192,257 were assembled into contigs (detailed information per sample is included in Supplementary Table S2). It is worth mentioning that after sequencing processing, around 60% of the reads were of bacterial origin. Thus, contamination with host DNA is a crucial element to consider when selecting the sequencing method and the depth of sequencing.

### Microbial Diversity and Structure of the Gut Microbiota

**FIGURE 2:**
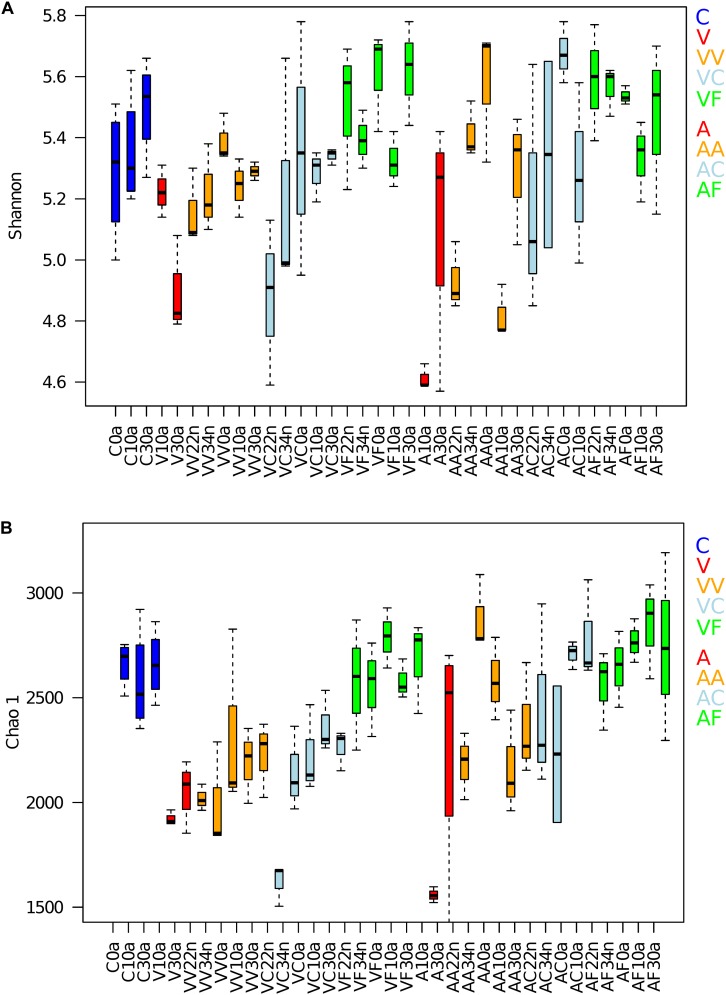
Alpha diversity of the gut microbiota. **(A)** Shannon index and **(B)** richness estimator (Chao1) were obtained for both groups (vancomycin and ampicillin) of experimental samples. C, control (4 samples per time point); V (3 and 4 samples at V10 and V30, respectively), G1 vancomycin treated; VV (3 samples per time point), G2 vancomycin treated; VC (3 samples per time point), G2 vancomycin free; VF (3 samples per time point), G2 vancomycin free + feces; A (3 and 4 samples at A10 and A30, respectively), G1 ampicillin treated; AA (3 samples per time point), G2 ampicillin treated; AC (3 samples per time point), G2 ampicillin free; and AF (3 samples per time point), G2 ampicillin free + feces.

The Shannon index (Shannon, 1948) and the richness estimators Chao1 (Chao, 1984) were calculated for each time point (Figure 2) and the values compared with the Wilcoxon signed-rank test. We compared the alpha-diversity of the controls with the samples included in the groups V, VV, VC, VF and A, AA, AC, AF for the vancomycin and ampicillin experiments, respectively. The samples belonging to each group were compared as a whole to the control to assess if the average ranges of values were statistically different. For the Shannon index a significant drop in the values during the first treatment was observed for both antibiotics compared to the controls (V, *p*-value = 0.006 and A, *p*-value = 0.031). In the vancomycin experiment, the antibiotic treatment affected the microbial richness (based on the Chao1) that significantly decreased at G1 compared to the controls (V, *p*-value = 3.969E-05), and that remained lower than the control at G2 (VV, *p*-value = 3.129E-05), including the VC samples (VC, *p*-value = 7.708E-06). For the ampicillin experiment the richness estimator Chao1 was also significantly lower than the control only at G1 (A, *p*-value = 0.017). Interestingly, the VF and AF diversity and richness did not show significant differences compared to the controls in any case, and even reached higher values.

**FIGURE 3:**
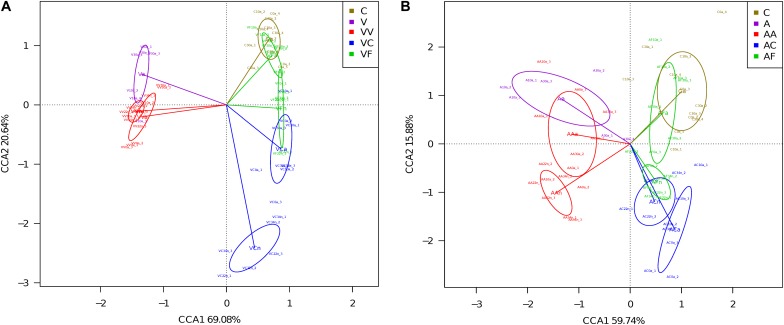
CCA showing microbiota composition clusters among the individuals analyzed. **(A)** Vancomycin experiment. **(B)** Ampicillin experiment. C, control; V, G1 vancomycin treated; VV, G2 vancomycin treated; VC, G2 vancomycin free; VF, G2 vancomycin free + feces; A, G1 ampicillin treated; AA, G2 ampicillin treated; AC, G2 ampicillin free; and AF, G2 ampicillin free + feces.

We carried out a CCA of microbial community composition from all individuals analyzed in each experiment (Figure 3). The 12 adult samples corresponding to the three control time points (C0a, C10a, and C30a) grouped together and non-significant differences were found between them (Adonis; *p*-value = 0.21). Therefore, for the comparisons, we have considered C0a, C10a, and C30a as a homogeneous population (named Ca). In the vancomycin experiment (Figure 3A), the first CCA axis explained 69.1% (the second axis 20.6%) of the overall variability (89.7%), separating clearly vancomycin-treated and non-treated samples. In the samples collected from vancomycin-treated individuals, two groups corresponding to the samples at G1 and G2 were obtained. In the G2 non-treated samples, those supplied with feces (VF) grouped closer with the controls than those without added feces (VC). When we applied the Adonis to compared whether the different groups (Ca, Va, VVa, VVn, VCa, VCn, VFa, and VFn) showed differences in composition, substantial and statistically significant differences were found (Adonis; *p*-value = 0.0017). In the ampicillin experiment (Figure 3B), a similar pattern can be observed, although the total represented variability (75.6%) is lower than in the vancomycin one. The first CCA axis separating ampicillin-treated from non-treated samples explained 59.7% (the second axis 15.8%) of the overall variability. As we did for the vancomycin, we applied the Adonis test to find global differences in composition associated with the different groups (Ca, Aa, AAa, AAn, ACa, ACn, AFa, and AFn) and also resulted in statistically significant differences (Adonis; *p*-value = 0.0017). The results indicate that antibiotics have a strong effect on the microbiota, but there is a resilience capacity since the diversity of the control microbiota could be restored (at least in part) by removing the antibiotic from the diet. Clearly, this recovery was faster, when the diet was supplemented with feces, with adults at 10 and 30 days at G2 achieving a similar composition to adults in the control population.

To further investigate the results obtained, we focused on the taxa with abundance levels above 0.1% in at least one of the 36 time point samples, both at phylum and family levels (see Supplementary Tables S3, S4).

#### Effect of Vancomycin or Ampicillin on the Gut Microbiota Composition

The outcome of the antibiotic treatment on the gut microbiota composition was different in both experiments (overview of composition in Supplementary Figure S1). We analyzed the differences obtained at family level comparing the control composition (Ca) with the other conditions by means of a Wilcoxon-signed rank test and the significant variable taxa were represented in heatmaps (Supplementary Figures S2, S3). Numerous taxa were significantly affected by both antibiotic treatments, although the number was higher in the case of vancomycin. Furthermore, more taxa differed in abundance at G2 than at G1, indicating the continuous effect of the antibiotics. In the vancomycin treated samples (Supplementary Figure S2 and Supplementary Table S4A) the main families showing a reduction in their relative abundance (colored red on the heatmaps) were Clostridiaceae, Lachnospiraceae, Clostridiales_uc, Ruminococcaceae (Firmicutes), Bacteroidaceae, Rikenellaceae, Prevotellaceae (Bacteroidetes), and Eggerthellaceae (Actinobacteria). Conversely, 10 families increased their abundance (colored blue on the heatmaps): Enterobacteriaceae, Yersiniaceae, Budviciaceae, Enterobacterales_uc, Orbaceae, Morganellaceae, Erwiniaceae, Vibrionaceae, Pectobacteriaceae (Proteobacteria), and Fusobacteriaceae (Fusobacteria). In the ampicillin treated samples (Supplementary Figure S3 and Supplementary Table S4B), we found three families, Odoribacteraceae, Porphyromonadaceae and Bacteroidaceae (Bacteroidetes), responding differently to the treatment, which could explain why large changes where not observed at phylum level. The abundance of the two former decreased at G2, whereas the latter one increased. In summary, when the antibiotics were administered to successive generations of insects, there were changes in the microbiota composition and this happened not only in the first, but also in the second generation.

#### Microbiota Recovery After Antibiotic Cessation

**FIGURE 4:**
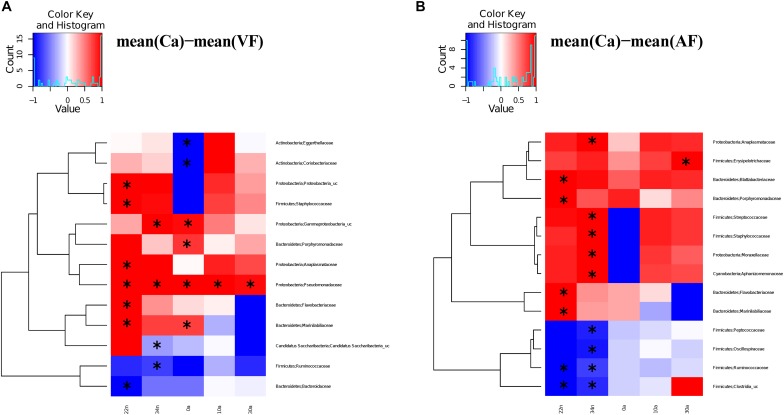
Significantly different taxa between controls and feces supplemented samples. Heatmaps representing the differences between the mean control composition (Ca) and the means of the rest of the conditions for those families presenting a statistically significant difference in at least one comparison. **(A)** Ca versus VF. **(B)** Ca versus AF. The statistical method used was the two-sample Wilcoxon rank-sum test with the Benjamini and Hochberg procedure to control the false discovery rate. To scale the data to [–1, +1] a normalization was performed by dividing the positive values with the maximum positive value and the negative values with the minimum negative value. Significant differences marked with an “^∗^”.

**FIGURE 5:**
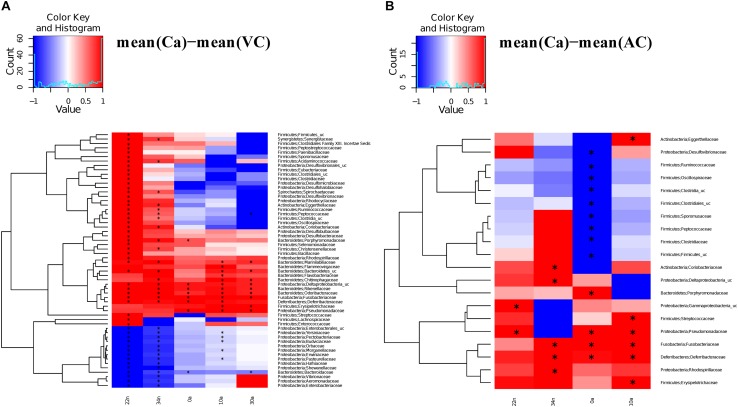
Significantly different taxa between controls and non-feces supplemented samples. Heatmaps representing the differences between the mean control composition (Ca) and the means of the rest of the conditions for those families presenting a statistically significant difference in at least one comparison. **(A)** Ca versus VC. **(B)** Ca versus AC. The statistical method used was the two-sample Wilcoxon rank-sum test with the Benjamini and Hochberg procedure to control the false discovery rate. To scale the data to [–1, +1] a normalization was performed by dividing the positive values with the maximum positive value and the negative values with the minimum negative value. Significant differences marked with an “^∗^”.

We compared the antibiotic free samples at G2 with the controls to find out if there was a partial or complete recovery of the microbiota composition after antibiotic cessation (VC), and with feces added to the diet (VF). For the vancomycin experiment, significant differences (Adonis test) were observed in the comparisons Ca *vs.* VC, both in nymphs (Ca *vs.* VC22n and VC34n, *p*-values < 0.01) and in adults (Ca *vs.* VC0a, *p*-value < 0.05; Ca *vs.* VC10a and VC30a, *p*-values < 0.01). However, when comparing Ca *vs.* VF only the differences at 22 day nymphal stage (Ca *vs.* VF22n, *p*-value < 0.01) and at adults day 0 was significant (Ca *vs.* VF0a, *p*-value < 0.05). Thus, the results indicated that there was no total recovery of the vancomycin treatment with adding feces until adults of 10 days. For the ampicillin experiment, in the comparisons Ca *vs.* VC only one significant difference was found at 0 days adults (Ca *vs.* AC0a, *p*-value < 0.01). When adding feces (Ca *vs.* AF), non-significant differences were obtained in nymphal and adult stage. Respect to the families affected by the treatment at G1, they tended, in general, to reach abundance values close to control population, recovery being faster when feces were added to the diet. VF and AF populations reached abundance values similar to controls and only a few taxa (13 and 14 in VF and AF, respectively) showed significant differences, mainly in nymphal stages (Figure 4 and Supplementary Table S4), giving support to the hypothesis that coprophagy is an important way for the offspring to acquire gut microbiota, as well as a mechanism for the maintenance and restoration of the microbiota when facing antibiotic perturbations. In the population without feces supplemented (VC and AC, Figure 5), we observed more taxa that differed significantly, although there was a tendency to recover some control values in adult samples.

#### Core Gut Microbiota Composition in Adults

The results obtained showing similar taxa composition in the second-generation adults (at 10 and 30 days) in both experiments when feces were added to the diet, and similar to that of control samples, indicate the existence of a resident gut microbiota. Therefore, we decided to analyze the taxa present in C10a, C30a, VF10a, VF30a, AF10a, and AF30a in all the samples and with an abundance value higher than 0.1% as indicative of the phyla and families that form the core of the gut microbiota of *B. germanica* adults (Supplementary Table S5). We decided not to include the time point 0a because this is the first time point after the last ecdysis, which affects the exoskeletal lining of the hindgut, eliminating attached bacterial populations, which requires time to reestablish stable microbiota. The most abundant phyla of the bacterial core were Bacteroidetes (64.6%), Firmicutes (15.2%), Proteobacteria (9.7%) and Fusobacteria (6.6%). These results agree with our previous studies of gut microbiota composition (Pérez-Cobas et al., 2015; Rosas et al., 2018). Nevertheless, in this work, we have identified other less abundant phyla as forming the core: Actinobacteria (0.4%), Spirochaetes (0.3%), Synergistetes (0.2%), and Verrucomicrobia (0.2%). In total, 41 families with abundances greater than 0.1% were present in the core gut microbiota. The most abundant were Porphyromonadaceae (28%), Bacteroidaceae (13%), Fusobacteriaceae (6.5%), Rikenellaceae (5.2%), Prevotellaceae (4.8%), Bacteroidales_uc (4.1%), Desulfovibrionaceae (4.1%), Ruminococcaceae (3.1%), Clostridiaceae (2.5%), Clostridiales_uc (2.4%), Lachnospiraceae (2.3%), Bacteroidetes_uc (2.3%), Flavobacteriaceae (1.6%), Odoribacteraceae (1.2%), and Sphingobacteriaceae (1%).

### Functional Analyses of the Gut Microbiota

We have carried out the functional analysis of the 113 metagenomes by comparison against the TIGRFAM database, obtaining a hierarchical classification for the main-roles (the highest functional level), sub roles (more specific metabolic functions for each one of the roles), and genes (metabolic functions) (Supplementary Table S8, respectively).

**FIGURE 6:**
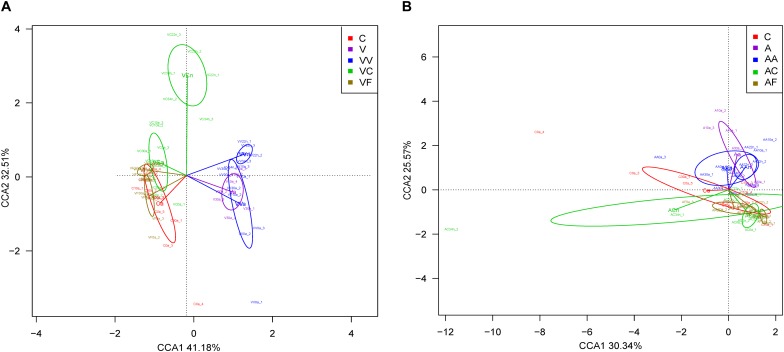
CCA based on genetic profile to compare the functional similarity of the experimental groups. **(A)** Vancomycin experiment. **(B)** Ampicillin experiment. C, control; V, G1 vancomycin treated; VV, G2 vancomycin treated; VC, G2 vancomycin free; VF, G2 vancomycin free + feces; A, G1 ampicillin treated; AA, G2 ampicillin treated; AC, G2 ampicillin free; and AF, G2 ampicillin free + feces.

We have carried out CCA based on the abundance of genes from all individuals analyzed in both experiments (Figure 6). The compared groups were the same than we compared in the taxonomic analysis: Ca, Va, VVa, VVn, VCa, VCn, VFa, and VFn for the vancomycin experiment and Ca, Aa, AAa, AAn, ACa, ACn, AFa, and AFn for the ampicillin one. The Adonis test to evaluate whether the grouping of the samples attributable to functional profiles is according to the treatment was significant in both CCA analyses (both *p*-values = 0.0017). In the vancomycin experiment (Figure 6A), both axes explained 73.7% of the overall variability, clearly separating vancomycin-treated (G1 and G2) samples on the one hand, nymphs at G2 fed without added feces (VC condition) in a second group, and the rest of the non-treated samples including controls at G1, all samples from the feces-supplemented population, and the VC adults. In the case of the ampicillin experiment (Figure 6B) both axes explained 55.9% of the overall variability, separating ampicillin-treated from non-treated samples, although the differences were not as clear as in vancomycin.

The main functional roles and their relative abundances are represented in Supplementary Figure S4 and Supplementary Table S6. In general, a great homogeneity in the distribution of the different time-point samples was observed in both experiments, with the exception of an intriguing shift in the relative abundance in the category of *mobile and extrachromosomal element functions* in both experiments, independently of the antibiotic treatment. This homogeneity indicated that main functional requirements were shared among the different bacterial communities, despite antibiotic treatment. However, some differences were observed in the subroles mainly due to the antibiotic treatment (Supplementary Table S7).

#### Effect of Antibiotics on the Genetic Potential of the Gut Microbiota

The only main role showing a slight increase for both antibiotic treatments is *transport and binding protein*. In vancomycin treated samples, *protein synthesis* and *DNA metabolism* showed a slight decrease at G1 and G2. Regarding sub roles, 53 showed significant differences, 37 of which were affected by comparing the control condition with antibiotic treated samples at G1 and G2 (Ca *vs.* Va; Ca *vs.* VVn; and Ca *vs.* VVa) (Table 1). Only seven were consistent in all three comparisons (independently of generation and developmental stage), five increased their abundance: *heme, porphyrin and cobalamin* (*biosynthesis of cofactors, prosthetic groups, and carriers*); *pathogenesis* (*cellular processes*); *carbohydrates, organic alcohols and acids, anions and nucleosides, purines and pyrimidines (transport and binding proteins*); and two decrease their abundance: *sporulation and germination* (*cellular processes*) and *tRNA aminoacylation* (*protein synthesis*). Furthermore, five sub roles were overrepresented (*p*-value < 1E-04) in some samples: *toxin production and resistance* (*cellular processes*); *nitrogen metabolism* and *sulfur metabolism* (*central intermediary metabolism*); *anaerobic and Entner-Doudoroff* (*energy metabolism*) and only one, *DNA replication, recombination and repair* (*DNA metabolism*) was underrepresented in one comparison.

**TABLE 1:** Sub role relative abundance comparison between pairs of groups in the vancomycin experiment.

**Main role**	**Sub role**	**Ca/Va**	**Ca/VVn**	**Ca/VVa**	**Ca/VCn**	**Ca/VCa**	**Ca/VFn**	**Ca/VFa**
Amino acid biosynthesis	Pyruvate family	NS	NS	NS	NS	NS	↓6.9E-3	NS
Biosynthesis of cofactors, prosthetic groups, and carriers	Biotin	↓1.6E-4	↓1.1E-4	NS	↓4.3E-4	NS	NS	NS
	Chlorophyll and bacteriochlorophyll	NS	↓1.1E-4	NS	NS	NS	NS	NS
	Folic acid	NS	NS	↑4.6E-4	NS	NS	NS	NS
	Heme, porphyrin, and cobalamin	↓4.0E-5	↓1.1E-4	↓6.8E-6	NS	NS	NS	NS
	Menaquinone and ubiquinone	NS	↓2.2E-4	NS	↓1.1E-4	NS	NS	NS
	Molybdopterin	↓5.2E-3	↓4.3E-4	NS	NS	NS	NS	NS
	Pyridoxine	NS	NS	NS	↑2.0E-3	NS	NS	NS
	Riboflavin, FMN, and FAD	NS	NS	NS	↑3.2E-3	NS	NS	NS
Cell envelope	Biosynthesis and degradation of murein sacculus and peptidoglycan	↑2.6E-3	NS	NS	NS	NS	NS	NS
	Other	↑1.6E-4	NS	NS	↓3.2E-3	NS	NS	NS
Cellular processes	Conjugation	NS	NS	NS	↑1.2E-3	NS	NS	NS
	Detoxification	NS	NS	NS	↓2.2E-3	NS	NS	NS
	Pathogenesis	↓4E-5	↓1.1E-4	↓6.8E-6	NS	NS	NS	NS
	Sporulation and germination	↑7.5E-4	↑3.2E-3	↑4.6E-4	NS	NS	NS	NS
	Toxin production and resistance	↓4.0E-5	↓1.1E-4	NS	↓1.1E-4	NS	NS	NS
Central intermediary metabolism	Nitrogen fixation	↓2.8E-4	↓4.3E-4	NS	NS	NS	NS	NS
	Nitrogen metabolism	↓4.0E-5	↓1.1E-4	NS	↓1.1E-4	NS	NS	NS
	Polyamine biosynthesis	↑4.8E-4	NS	NS	NS	NS	NS	NS
	Sulfur metabolism	↓4.0E-5	↓4.3E-4	NS	NS	NS	NS	NS
DNA metabolism	Chromosome-associated proteins	NS	NS	NS	↓1.1E-4	NS	NS	NS
	DNA replication, recombination, and repair	NS	NS	↑6.8E-6	NS	NS	NS	NS
Energy metabolism	Anaerobic	NS	↓1.1E-4	↓4.8E-5	NS	NS	NS	NS
	Biosynthesis and degradation of polysaccharides	NS	NS	NS	↓1.1E-4	NS	NS	NS
	Chemoautotrophy	NS	NS	NS	↑1.1E-4	NS	NS	NS
	Electron transport	NS	↓1.1E-4	NS	NS	NS	NS	NS
	Entner-Doudoroff	NS	↓1.1E-4	↓8.2E-5	NS	NS	NS	NS
	Methanogenesis	NS	NS	NS	↑3.2E-3	NS	NS	NS
	Pyruvate dehydrogenase	NS	NS	NS	↑2.0E-3	NS	NS	NS
	Sugars	↓2.8E-4	↓1.1E-4	NS	↓1.1E-4	NS	NS	NS
Fatty acid and phospholipid metabolism	Biosynthesis	NS	↓4.3E-4	NS	NS	NS	NS	NS
	Degradation	NS	↓4.3E-4	NS	NS	NS	NS	NS
Mobile and extrachromosomal element functions	Plasmid functions	NS	↓9.7E-3	↓7.3E-3	NS	NS	NS	NS
Protein fate	Protein and peptide secretion and trafficking	↓4.8E-4	↓1.1E-4	NS	↓1.1E-4	NS	NS	NS
	Protein folding and stabilization	↑2.6E-3	NS	NS	↑4.7E-3	NS	NS	NS
Protein synthesis	Other	NS	NS	↑6.6E-4	↑9.7E-3	NS	NS	NS
	Ribosomal proteins: synthesis and modification	NS	NS	NS	↑4.3E-4	NS	NS	NS
	Translation factors	NS	NS	NS	↑7.5E-4	NS	NS	NS
	tRNA aminoacylation	↑2.6E-3	↑3.2E-3	↑6.6E-4	↑3.2E-3	NS	NS	NS
Purines, pyrimidines, nucleosides, and nucleotides	Pyrimidine ribonucleotide biosynthesis	NS	↑3.2E-3	↑6.6E-4	↑2.0E-3	NS	NS	NS
	Salvage of nucleosides and nucleotides	NS	↑6.9E-3	↑2.0E-4	NS	NS	NS	NS
Regulatory functions	DNA interactions	NS	↓4.3E-4	NS	NS	NS	NS	NS
	Other	NS	↑4.7E-3	NS	NS	NS	NS	NS
Signal transduction	PTS	NS	↓4.7E-3	NS	NS	NS	NS	NS
	Two-component systems	NS	NS	NS	↓1.1E-4	↓8.2E-5	NS	NS
Transcription	Degradation of RNA	NS	NS	NS	↑1.1E-4	NS	NS	NS
	Transcription factors	NS	↑4.7E-3	NS	NS	NS	NS	NS
Transport and binding proteins	Amino acids, peptides and amines	↓2.8E-4	↓4.3E-4	NS	NS	NS	NS	NS
	Anions	↓7.5E-4	↓1.1E-4	↓9.4E-4	NS	NS	NS	NS
	Carbohydrates, organic alcohols, and acids	↓1.6E-4	↓1.1E-4	↓4.8E-5	NS	NS	NS	NS
	Nucleosides, purines and pyrimidines	↓1.6E-4	↓1.1E-4	↓1.3E-4	↓2.2E-4	NS	NS	NS
	Porins	NS	NS	NS	↓2.0E-3	NS	NS	NS
	Unknown substrate	NS	NS	NS	↓1.3E-3	NS	NS	NS

*Only significant sub roles in at least one comparison are shown (*p*-value < 0.05 and 90% confidence interval that do not overlap between the pairwise comparison). Upward and downward arrows indicate those sub roles that were more and less abundant, respectively, in the first of the two conditions under comparison. NS, not significant.*

As in the taxonomic analysis, the effect of the ampicillin seems to be less strong than the vancomycin one. Non-consistent changes at main role level were observed, except the already mentioned in *transport and binding proteins*. At sub role level, three changed significantly in the first generation as a direct response to the antibiotic (Ca *vs.* Aa), and seven in nymphs in the second generation (Ca *vs.* AAn). However, there were no consistent changes in the two treated generations. Moreover, non-significant changes were found in treated second-generation-adults (Ca *vs.* AAa) (Table 2).

**TABLE 2:** Sub role relative abundance comparison between pairs of groups in the ampicillin experiment.

**Main role**	**Sub role**	**Ca/Aa**	**Ca/AAn**	**Ca/AAa**	**Ca/ACn**	**Ca/ACa**	**Ca/AFn**	**Ca/AFa**
Cellular processes	Pathogenesis	↓2.8E-4	NS	NS	NS	NS	NS	NS
	Sporulation and germination	NS	↑3.2E-3	NS	NS	NS	↓1.1E-4	NS
	Toxin production and resistance	NS	↓1.1E-4	NS	NS	NS	NS	NS
Central intermediary metabolism	Phosphorus compounds	NS	↓7.5E-4	NS	NS	NS	NS	NS
DNA metabolism	Chromosome-associated proteins	NS	↓1.1E-4	NS	NS	NS	NS	NS
Energy metabolism	Pyruvate dehydrogenase	↑1.2E-3	NS	NS	NS	NS	NS	NS
	Sugars	NS	↓2.2E-4	NS	NS	NS	NS	NS
Mobile and extrachromosomal element functions	Other	NS	NS	NS	NS	NS	↑4.7E-3	NS
Protein fate	Degradation of proteins, peptides, and glycopeptides	NS	↑3.2E-3	NS	NS	NS	NS	NS
Regulatory functions	DNA interactions	NS	NS	NS	NS	NS	↓2.0E-3	NS
Signal transduction	Two-component systems	NS	NS	NS	NS	NS	↓4.3E-4	NS
Transport and binding proteins	Amino acids, peptides and amines	NS	NS	NS	NS	NS	↓9.7E-3	NS
	Carbohydrates, organic alcohols, and acids	NS	↓4.3E-4	NS	NS	NS	NS	NS
	Cations and iron carrying compounds	NS	NS	NS	NS	NS	↓4.3E-4	NS
	Porins	↓7.5E-4	NS	NS	NS	NS	NS	NS
Unknown function	Enzymes of unknown specificity	NS	NS	NS	NS	NS	↓4.3E-4	NS

*Only significant sub role differences in at least one comparison are shown (*p*-value < 0.05 and 90% confidence interval that do not overlap between the pairwise comparison). Upward and downward arrows indicate those sub roles that were more and less abundant, respectively, in the first of the two conditions being compared. NS, not significant.*

#### Functional Recovery After Antibiotic Cessation

An important finding of the taxonomic analysis was the almost total recovery of the main taxa in adults at G2 when feces were added to the diet. As we did for the bacterial composition, we compared the functions (gene abundance) between groups with the Adonis test. The vancomycin experiment showed that at functional level all the comparisons of the control with the adult samples with (Ca *vs.* VC) and without feces (Ca *vs.* VF) were not significant. In the case of the nymphs, the comparisons were significant in samples without added feces (Ca vs. VC22n and VC34n, *p*-values < 0.01) but not significant in the samples with added feces. Then, we compared the relative abundance of the subroles in nymphs and adults (VCn, VCa, VFn, and VFa) with the control (Ca) (Table 1). When comparing the sub roles of the control with the fecal-supplemented samples in both nymphs (Ca *vs.* VFn) and adults (Ca *vs.* VFa), only one difference in the *pyruvate family* (*amino acid biosynthesis*) was found in nymphs (Table 1). An unexpected result was obtained in the sub roles in the samples fed only diet (VC). The comparison with the nymphs (Ca *vs.* VCn) highlighted 27 significantly different sub roles, whereas in adults (Ca *vs.* VCa) recovery was complete with only one exception in the two component systems (*signal transduction*), which suggests that the control functions can be achieved in adults without fecal supplementation. In the ampicillin experiment, the Adonis test was non-significant in any of the comparisons, both adults and nymphs samples with or without feces. Non-significant differences were found in adult stage in the non-treated samples with and without fecal supplementation (Ca *vs.* AFa and Ca *vs.* ACa) (Table 2). However, nymphs treated with feces (Ca *vs.* AFn) showed seven differences, and those of the non-supplemented diet population (Ca *vs.* ACn) lacked significant differences. This result should be taken with caution as despite the AC population was funded as the other ones, all individuals of ACn and ACa came from the same female, and no individual reached the 30 days adult stage, as it was indicated in section “Materials and Methods.”

#### Functions Core of Gut Microbiota in Adults

Overall, the results observed showed functional redundancy in the microbiota of *B. germanica*. We decided to determine the core functions of adults (Supplementary Table S9), based on the same samples that we used to define a core microbiota (C10a, C30a, VF10a, VF30a, AF10a, and AF30a). We found a wide repertoire of metabolic processes, including *transport and binding proteins, energy metabolism* (containing metabolism of sugars, amino acids and polysaccharides), *protein synthesis, DNA metabolism*, *protein fate*, and *regulatory functions*. Furthermore, pathways related to the synthesis of vitamins were identified, such as biosynthesis of vitamin B9 (folic acid), B1 (thiamin), B12 (cobalamin), K_2_ (menaquinone), or biotin. *Nitrogen fixation* and *metabolism* was also identified in the metagenomes. It is worth mentioning that one of the most abundant sub roles was related to *mobile and extrachromosomal element functions*.

#### Diversity of Antibiotic Resistance Genes

In order to find whether there was any change in the abundance of antibiotic resistance genes (ARGs) as a consequence of antibiotic treatment, we have compared all reads against the ARDB in the control and in vancomycin and ampicillin treated samples. For this preliminary survey, we considered those genes present in at least two of the biological replicates in at least two of each grouped sampling time as genes representative of controls and antibiotic-treated samples (Supplementary Table S10). Ten different antibiotic resistance genes were identified distributed as follows: four in control samples, three in Va, seven in VVn and VVa, one in Aa and seven in AAn and AAa samples. In the control (Ca), the genes *acrB*, *bacA*, *mexB*, and *tetM* could confer resistance to different antibiotics like aminoglycoside, glycylcycline, β-lactam, macrolide, acriflavin, bacitracin, tigecycline, fluoroquinolone, and tetracycline.

The vancomycin treated samples did not harbor a resistance gene for vancomycin, in neither the first (Va) or in the second generation (VVn and VVa). The genes detected in controls were also detected in Va (V10a and V30a), with the exception of *tetM*. In nymphal stage VVn (VV22n and VV34n samples), in addition to the three ARGs detected in Va and in the control, four new ARGs were detected: *macB, mdtK*, *rosA*, and *smeE*, which presumably confer the same resistance profile as the control condition besides enoxacin, norfloxacin, and fosmidomycin. However, in adults (VVa) the genes *macB* and *mdtK* were not detected. In the ampicillin treated first-generation samples Aa (A10a and A30a), only the gene *bacA* conferring resistance to bacitracin was detected. However, in the second generation we observed an increase in the number of ARGs. In nymphal stages (AAn) we detected four genes *acrB*, *bacA*, *mexB* also detected in control, and *ksgA*, which confer resistance to kasugamycin. In the adult stage (AAa), six ARGs were detected, two (*macB* and *rosB*) more than in the control condition.

When the ARGs were assigned to a putative phylum, all of them were detected in the Proteobacteria, with one exception, *tetM* gene, assigned to Bacteria_uc or Firmicutes. Most of ARGs in antibiotic treated samples are multidrug resistance efflux pumps. Furthermore, the ARGs detected seemed not to respond to the selective pressure produced by the antibiotic treatment administrated, but reflected taxonomic level changes, therefore the surviving bacteria. Further studies and analyses will be necessary to deep on this important topic.

### Effect of Antibiotics on *Blattabacterium*

We estimated the average number of copies of *ure*C gene from *Blattabacterium* at all time points at G1 and G2. Non-significant differences were obtained, independently of whether or not samples were treated with vancomycin or ampicillin. The average copy number of the *actin*5C gene from the host, used as a control, did not show significant changes (Supplementary Figure S5). Thus, *Blattabacterium* is not affected by either vancomycin or ampicillin, at least at the concentration used in this work (0.02%).

## Discussion

*B. germanica* is an omnivorous insect in which two symbiotic systems coexist in each individual, *Blattabacterium* in the fat body and the microbiota in the hindgut. *Blattabacterium* has a role in the provision of essential amino acids and participation in the nitrogen metabolism (López-Sánchez et al., 2009), but little is known about the role of the gut microbiota in host physiology. In this work, we aimed to disturb the gut microbiota with two antibiotics: vancomycin that acts against Gram-positive bacteria and thus not affecting the endosymbiont, and the broad spectrum antibiotic ampicillin that act against Gram-positive and some Gram-negative bacteria. We examined whether the treatments affected *Blattabacterium*, finding that it was not significantly affected by either of the antibiotics. Then, we evaluated the intergenerational impact of two types of antibiotics on the gut microbiota composition and functions based on metagenomics. We found that the community structure was strongly disturbed by the intake of both antibiotics, with vancomycin demonstrating a greater effect. The different effects observed between vancomycin and ampicillin may be due to the antibiotic efficiency, its action spectrum, or the doses employed. To date, the effects of three antibiotics have been studied in *B. germanica* at the same doses (0.02%), and rifampicin appears to affect the bacterial community most drastically (Rosas et al., 2018). Vancomycin treatment reduced Gram-positive bacteria (Firmicutes and Actinobacteria), but also diminished bacteria belonging to Bacteroidetes, indicating that some Gram-negative bacteria in this phylum are either sensitive to vancomycin, or to the effect on other ecologically dependent bacteria. A similar result has been found in mice and it was postulated that vancomycin (a non-absorbable antibiotic, at least in mice and humans) reached high enough concentrations in the gut to inhibit bacteria belonging to the Bacteroidetes phylum. Thus, some bacteria such as Proteobacteria and Fusobacteria take advantage of the empty niche to overgrow (Ubeda and Pamer, 2012; Vrieze et al., 2014). At phylum level, major changes were not observed in ampicillin-treated samples, except for a slight increase in the Bacteroidetes/Firmicutes ratio. An increase in the Bacteroidetes/Firmicutes ratio was also found by Panda et al. (2014) working with fecal microbiota of humans treated with fluoroquinolones and beta-lactams to study the short-term effect of antibiotics.

At a functional level, both antibiotics have a significant impact but stronger in the vancomycin experiment compared to the ampicillin one. Vancomycin led to alterations in the sub role *carbohydrates, organic alcohols and acids* (*transport and binding protein*). Similar disturbances in this function have been described in the gut microbiota of mice and humans treated with antibiotics (Ng et al., 2013; Pérez-Cobas et al., 2013, 2014; Theriot et al., 2014). The same main roles that were most abundant in the gut microbiota of *B*. *germanica* (*transport and binding proteins*, *energy metabolism*, and *protein synthesis*) were the most abundant in a metagenome analysis of the human gut microbiota (Pérez-Cobas et al., 2013). Because these are basic functions of a living microbe, it would explain why they are shared by the human and the cockroach gut microbiota, although it cannot be ruled out that it is due to the nutritional similarities in both omnivorous diets.

Coprophagy is a major force shaping gut microbiota in gregarious insects, such as cockroaches (reviewed in Onchuru et al., 2018). We have found that in *B. germanica* coprophagy leads to convergence toward a similar microbiota composition and functions during development, independently of whether the nymphs founding the populations were offspring of mothers treated with vancomycin or ampicillin. No significant differences were detected between VF and AF samples during development, which ended up displaying practically the same taxa in 10- and 30-day-old adults in the second generation and similar to the control samples in adults, indicating that a similar environment breeds a stable adult core microbiota. The main phyla forming the bacterial core (Bacteroidetes, Firmicutes, Proteobacteria, and Fusobacteria) were the ones described in previous studies of gut microbiota composition in *B. germanica* (Pérez-Cobas et al., 2015; Kakumanu et al., 2018; Rosas et al., 2018), although Kakumanu et al. (2018) did not find Fusobacteria among the most abundant phyla in lab-reared *B*. *germanica*. In this work, we have also detected other less abundant phyla as forming the core of adults: Actinobacteria, Spirochaetes, Synergistetes, and Verrucomicrobia, indicating the complexity of the gut microbiota in cockroaches. In the last years, the study of the microbiota of different animal species has shown that they harbor different kinds of gut communities, both in terms of abundance and composition (reviewed in Moran et al., 2019). Comparing the main phyla, *B. germanica* shares Bacteroidetes and Firmicutes with other omnivorous cockroaches (*Periplaneta americana* and *S. lateralis*) (Schauer et al., 2014; Tinker and Ottesen, 2016), and with more distantly related animals, such as mammals that are also omnivorous (Vandeputte et al., 2017). On the other hand, Proteobacteria and Fusobacteria are shared with its close relatives, the wood-feeding termites (Brune and Dietrich, 2015), whereas Proteobacteria and Firmicutes are the common phyla shared with different lineages of Lepidoptera (Hammer et al., 2017).

The importance of feces in *B. germanica* gut microbiota transmission and equilibrium at adult stage is even more evident if we look at the functional profiles. Adults in the second antibiotic-free generation with a feces-supplemented diet (VFa and AFa) proved similar to control-population adults (Ca) than to the antibiotic affected insects. Moreover, nymphs also tended to have similar functions, at least in one population (VFn), with only significant differences in amino acid biosynthesis. This could indicate that in similar environments, like that of the lab-reared cockroaches, the bacterial community of *B. germanica* is assembled to reach a characteristic composition, and functions, some of which might be those required by the host. The convergence for essential functions has been described in the hindgut paunch of *Amitermes wheeleri* collected from cow dung and in *Nasutitermes corniger* feeding on sound wood. In this study, the authors found community divergence in both species but similar functions were performed, such as hydrolytic enzymes, homoacetogenesis and cell motility and chemotaxis (He et al., 2013). Future studies, working with germ-free cockroaches, and with antibiotics acting against *Blattabacterium* (i.e., rifampicin) would help us to disentangle the role of the microbiota and if there is a crosstalk between the two symbiotic systems.

The main roles of gut microbiota in *B. germanica* were conserved even in the antibiotic treated-samples, suggesting a buffering capacity of the microbiota to perform essential functions. Functional redundancy must be essential to guarantee the function of the system even in the face of a disturbance, since other microorganisms can take over the functions carried out by those affected by the perturbation (Foster et al., 2017). This could occur more easily in species-rich microbiota, such as cockroaches, than in species-poor microbiota, such as *Drosophila melanogaster* or social bees (Broderick and Lemaitre, 2012; Kwong and Moran, 2016), since multiple taxa can be equivalent with respect to a given function. As cockroaches are omnivores, the taxa and the specialized functions they perform must be related with their omnivorous diet. For example, the most abundant gene of the *transport and binding proteins* encode for a TonB-linked outer membrane protein (SusC/RagA family) belonging to the Bacteroidetes phylum. This protein participates in the metabolism of proteins and carbohydrates (Cho and Salyers, 2001), possibly contributing to host food digestion. In addition, different pathways from the metagenomes related to energy production, including amino acids and carbohydrates metabolism, could be related to the host diet digestion. As it occurs in the human and other animals’ gut, we identified in this insect gut microbiota the capacity of synthetize vitamins (Rowland et al., 2018). A previous study of *B. germanica* found gut bacteria that could be associated to *nitrogen fixation* and *metabolism* (Pérez-Cobas et al., 2015). Here, we confirmed the presence of these capacities in the gut metagenomes of this cockroach. Interestingly, from the *transport and binding proteins* category, other abundant genes are encoding for the MFP subunit of efflux transporter (RND family) and the MATE efflux family protein, both systems related to antibiotic and drug resistance. Moreover, processes related to *mobile and extrachromosomal element functions*, including *prophage* and *plasmid functions* are also abundant in the gut microbiota of this animal. Thus, much like humans, cockroaches harbor a repertoire of resistance genes and spreading mechanisms in their gut microbiota.

We have found that *B. germanica* microbiota harbors ARGs in laboratory control conditions, and thus we can postulate that natural populations also harbor a resistance gene repertoire (resistome) in their microbiota. It is well known that one factor inducing the spread of antibiotic resistant bacteria is the systematic used of antibiotic in humans that can have transformed the gut microbiota into a reservoir of ARGs (Pérez-Cobas et al., 2013). Although we expected an increase in the abundance of those ARGs against the specific antibiotic used, we failed to detect a vancomycin resistance gene in the vancomycin-treated samples. Several possible explanations, not mutually exclusive, could be invoked to explain this fact. It is possible that the lack of antibiotic pressure in the lab-reared population for over 30 years and the inability to obtain new ARGs from an external environment could have reduced the variety of ARGs in the control condition samples. Besides, the Gram-positive bacteria lacking ARGs for vancomycin might be protected inside a biofilm due to the absorption of antibiotic with components of the biofilm, the reduced penetration of the antibiotic, the high bacterial density and/or the slower growth of the bacteria in the biofilm (Stewart and Costerton, 2001). Furthermore, the resistance to vancomycin could be due to specific SNPs (for example in the genes *sarA*, *vraR*, *vraS*, etc.) rather than to ARGs (Alam et al., 2014; Yamaguchi et al., 2019). Finally, the resistance of the survived bacteria could also be due to novel ARGs with homologs lacking in the databases and thus undetected. In the case of ampicillin, we detected some ARGs that can confer resistance to beta-lactam antibiotic, but the resistance induced by this antibiotic treatment also increased other types of resistance. In both antibiotic experiments, the Proteobacteria phylum had the highest variability of resistance types and most of these ARGs are multidrug resistance efflux pumps that confer cross-resistance to different antibiotics, since it can actively extrude a variety of compounds (Blanco et al., 2016).

A striking result is that the microbiota composition and functions were almost recovered in adults in VC and AC samples. As it was demonstrated that nymphs are born sterile, with the exception of *Blattabacterium* (Carrasco et al., 2014), and no feces from control population were supplied to VC and AC populations, these results could indicate that the microbiota can also be acquired from the environment, which would include non-sterile food, plastic jars, etc. We cannot rule out the possibility of contact with the mother’s feces until isolation of newborn nymphs (at most 24 h after hatching). In future research, we will work with a sterile environment to evaluate its real effect.

The results clearly indicate that regardless of the antibiotic perturbation to the microbiota in one generation, once such disturbance agent disappears, the microbiota composition and functions tend to recover in adults in the following generation, and in a natural and faster way with feces consumption. The complete restoration of the microbiota indicates that *B. germanica* contains a stable core of bacterial species inhabiting the gut. The faster recovery of the microbiota when feces are consumed corroborates that coprophagy is an important mechanism of acquisition and transmission of the microbiota for this species. Futures studies based on germ-free cockroaches will be necessary to understand the putative role played by the environment in providing microbes to the gut of cockroaches. Finally, *B. germanica* is a pathogen transmission vector, including those that are carriers of resistance to a wide variety of antibiotics. Understanding its symbiotic system will help to better face this important human pest.

## Data Availability Statement

All the sequences of gut metagenomes in this study were submitted to the European Bioinformatics Institute (EBI), EMBL Nucleotide Archive under the study accession number PRJEB31051, with accession numbers (ERS3121362–ERS3121474).

## Author Contributions

RD-S, AM, CG-F, and AL design the experiment. RD-S, AP-C, AM, CG-F, and AL wrote the manuscript. RD-S carried out the laboratory work. CG-F and IT contributed to the laboratory work. AA, AP-C, RD-S, and AL analyzed the data. RD-S, AP-C, JC, and AM contributed to the data analysis. All authors edited the manuscript and approved the final draft.

## Conflict of Interest

The authors declare that the research was conducted in the absence of any commercial or financial relationships that could be construed as a potential conflict of interest.
