# Supplementary material for: Unraveling Assemblage, Functions and Stability of the Gut Microbiota of Blattella germanica by Antibiotic Treatment
Source: Front Microbiol. 2020 Mar 25;11:487. doi: 10.3389/fmicb.2020.00487 (PMC7109288; doi:10.3389/fmicb.2020.00487)
Supplement: Supplementary file 1 [file Data_Sheet_1.PDF]

SUPPLEMENTARY FIGURES

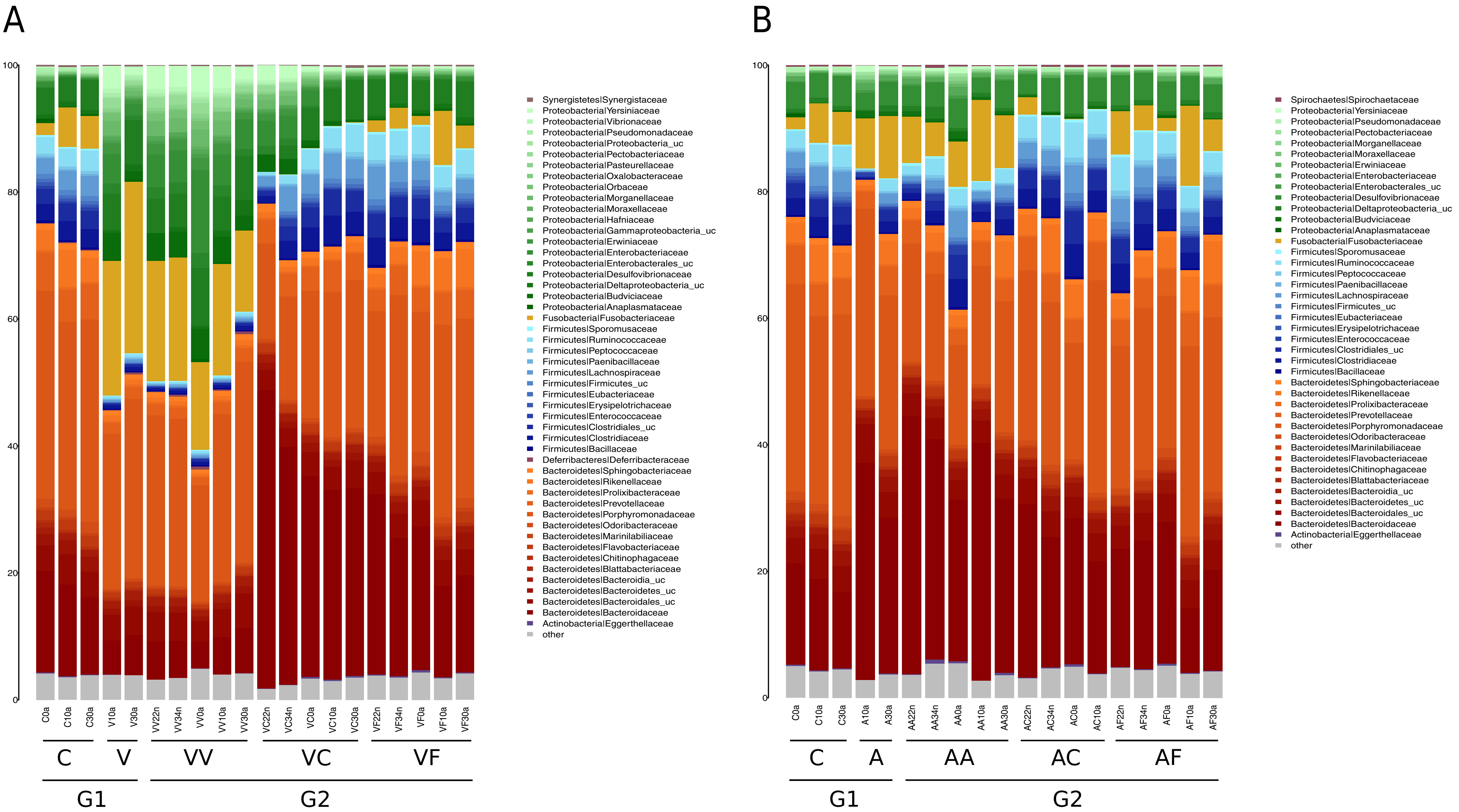

Supplementary Figure S1. Bacterial composition of the gut microbiota per sample. Relative abundance of major taxa found in the different time points in vancomycin (A) and ampicillin (B) experiments. C, control samples at G1. V, VV and A, AA vancomycin and ampicillin treated populations at G1 and G2, respectively. VC, VF and AC and AF non-antibiotic treated populations at G2. In VF and AF populations, feces for a lab-reared populations have been added. The three control samples (C0, C10 and C30) are the same for the two experiments. For a better resolution, only those taxa with an abundance higher than 0.4% in at least one of the time sample were included. See Figure 1 for further details.

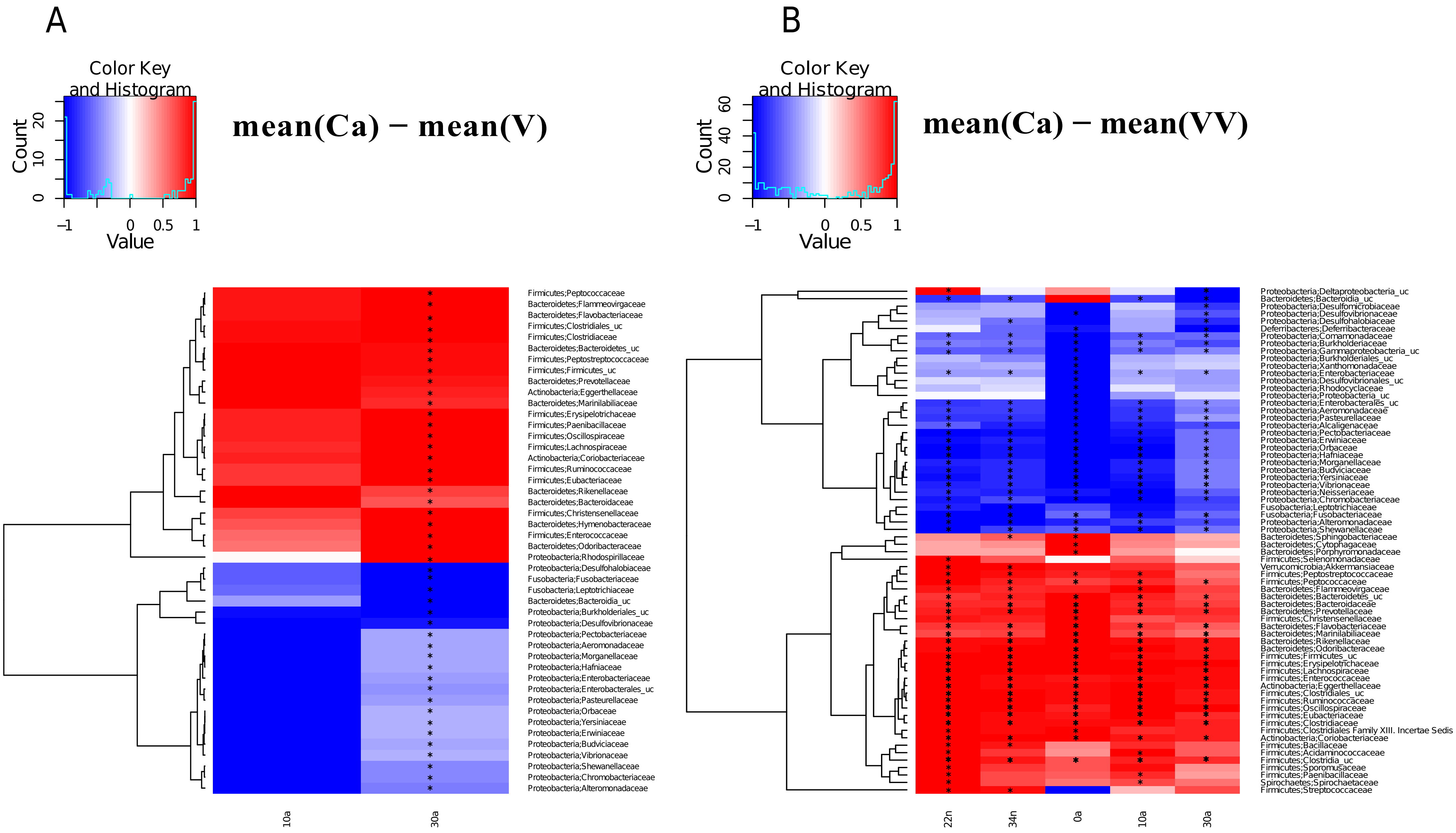

Supplementary Figure S2. Significantly different taxa between controls and vancomycin treated samples. Heatmaps representing the differences between the mean control composition (Ca) and the means of the rest of the conditions for those families presenting a statistically significant difference in at least one comparison. (A) Ca versus V; (B) Ca versus VV. The statistical method used was the two-sample Wilcoxon rank-sum test with the Benjamini & Hochberg procedure to control the false discovery rate. To scale the data to [-1, 1] a normalization was performed by dividing the positive values with the maximum positive value and the negative values with the minimum negative value. Significant differences marked with an “*”.

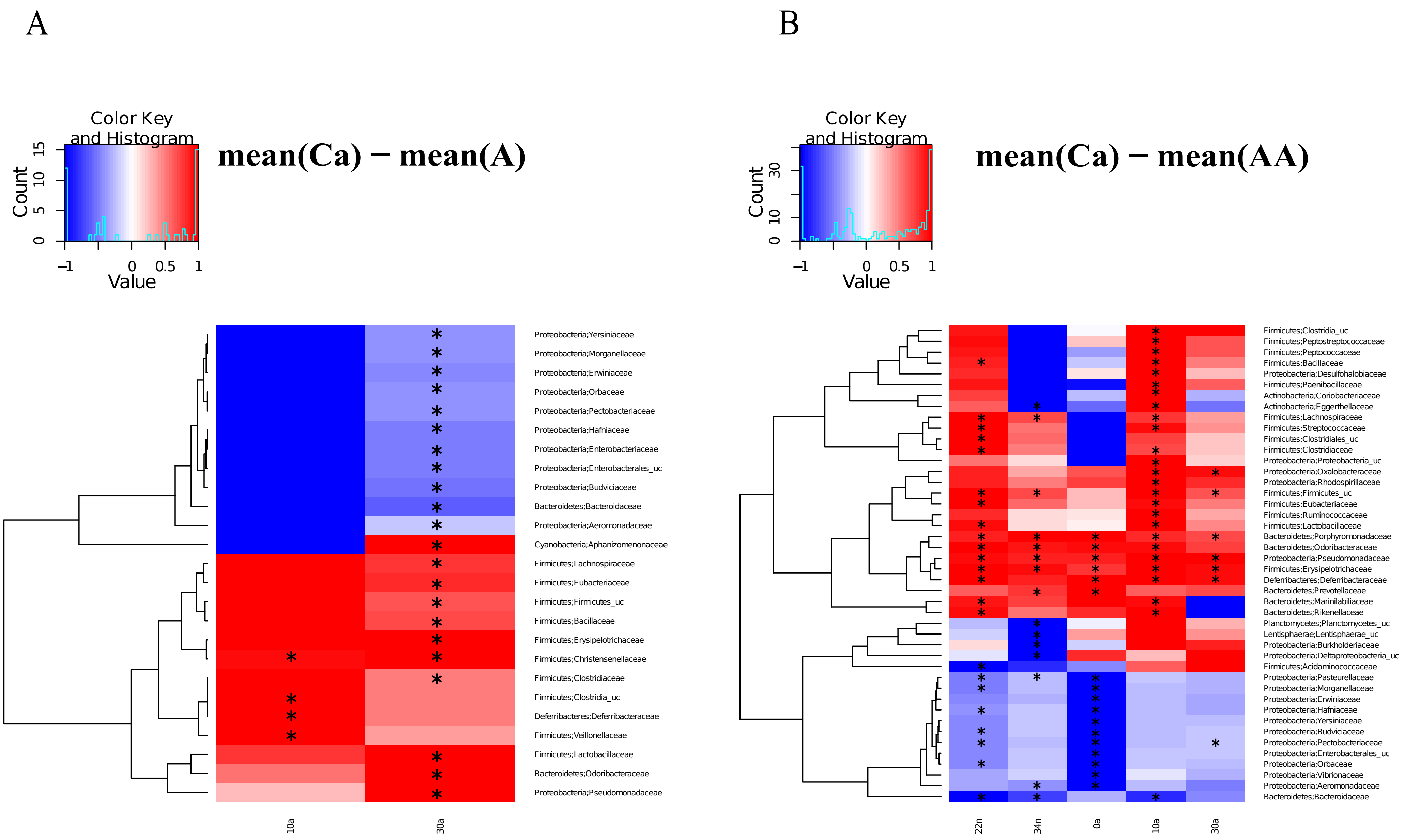

Supplementary Figure S3. Significantly different taxa between controls and ampicillin treated samples. Heatmaps representing the differences between the mean control composition (Ca) and the means of the rest of the conditions for those families presenting a statistically significant difference in at least one comparison. (A) Ca versus A; (B) Ca versus AA. The statistical method used was the two-sample Wilcoxon rank-sum test with the Benjamini & Hochberg procedure to control the false discovery rate. To scale the data to [-1, 1] a normalization was performed by dividing the positive values with the maximum positive value and the negative values with the minimum negative value. Significant differences marked with an “*”.

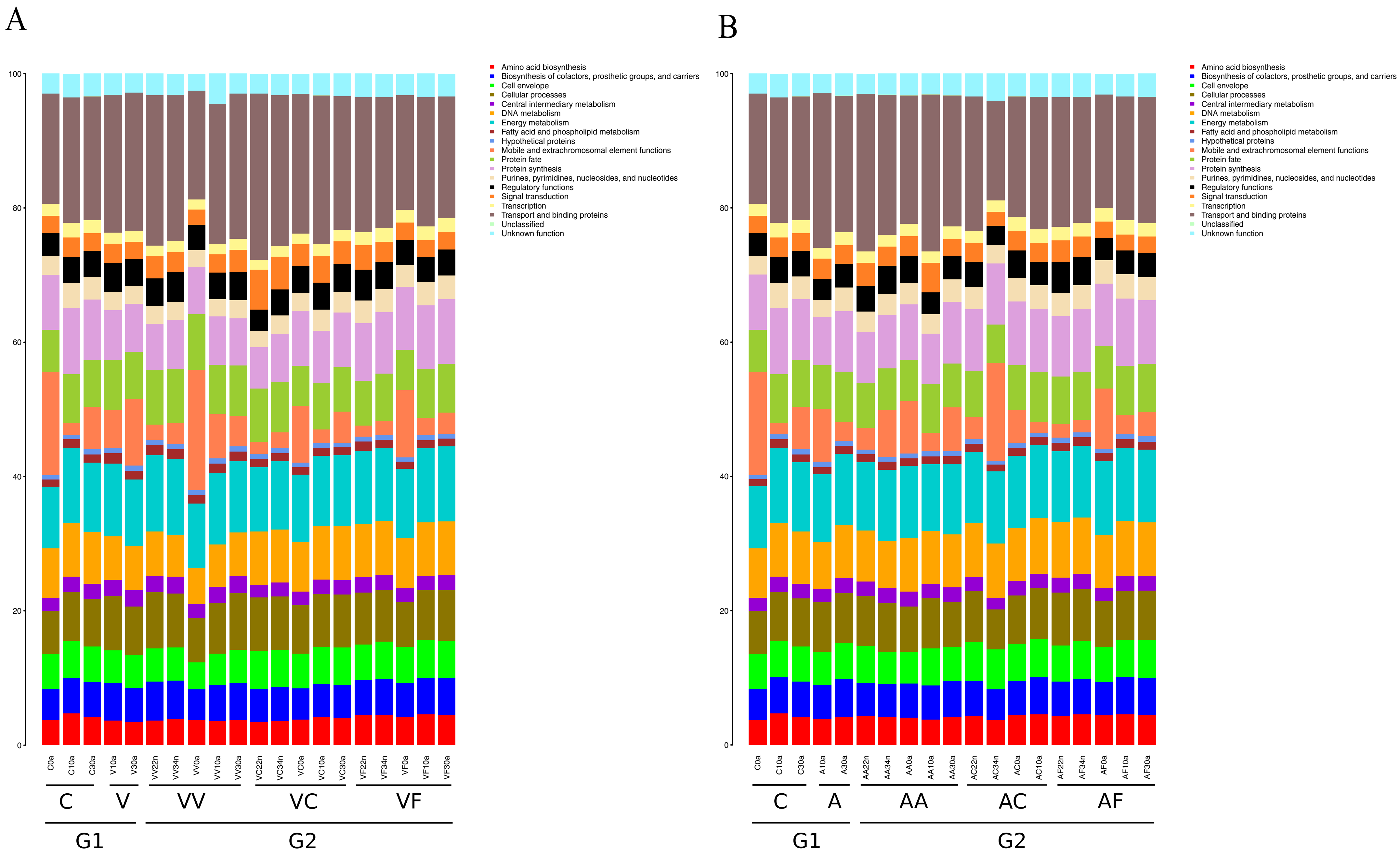

Supplementary Figure S4. Relative abundance of main functional roles in the different time points. A) Vancomycin experiment and B) ampicillin experiment. C, control samples at G1. Rest as in Figure S1.

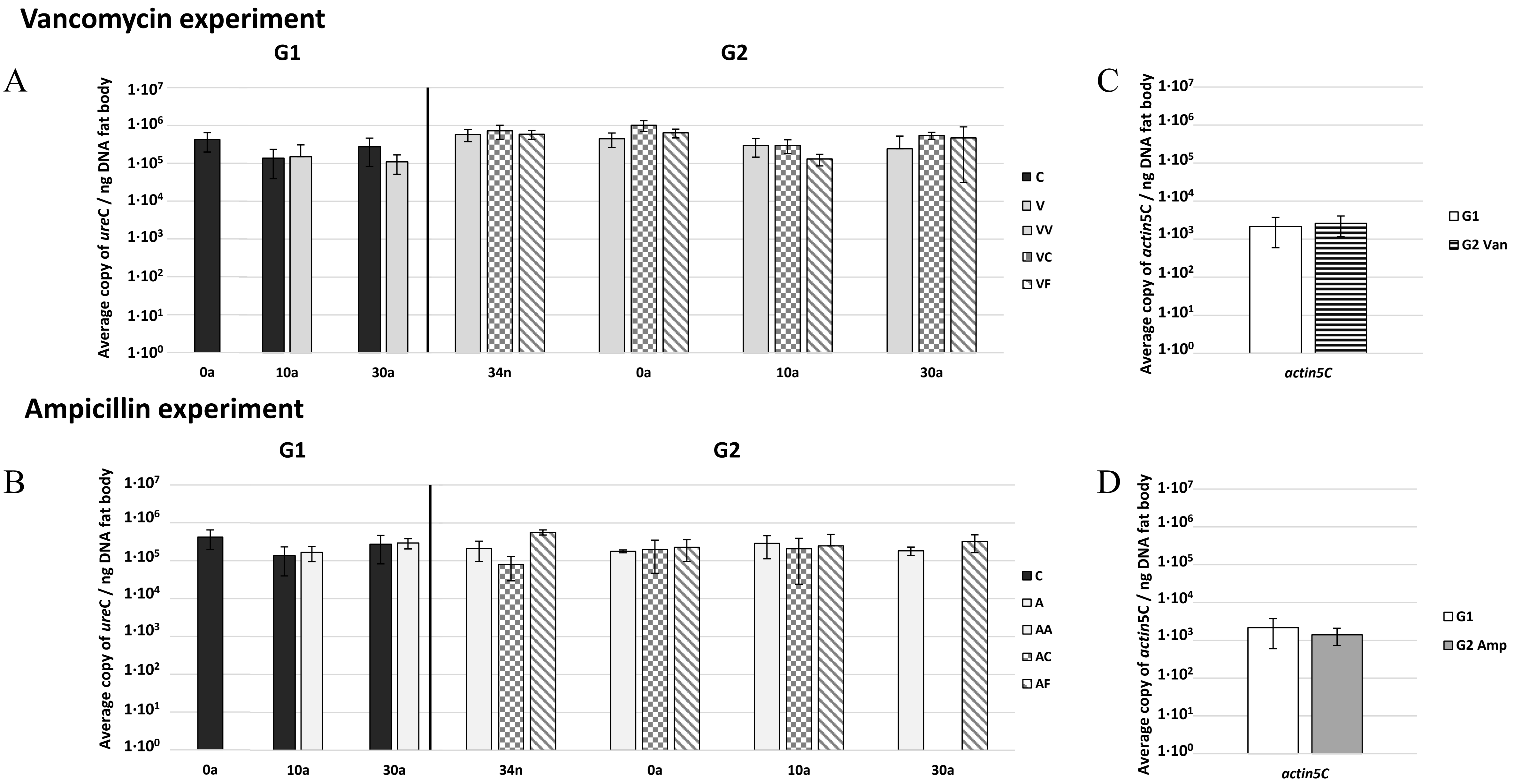

Supplementary Figure S5. Quantification of *Blattabacterium* population in fat body of cockroaches. Average copy number of *ure*C per ng DNA in samples of vancomycin (A) and ampicillin (B) experiment at the different time points of G1 and G2. Average copy number of *actin*5C per ng DNA fat body in samples of G1 and G2 in vancomycin (C) and ampicillin (D) experiments.
