# Supplementary material for: Unraveling Assemblage, Functions and Stability of the Gut Microbiota of Blattella germanica by Antibiotic Treatment
Source: Front Microbiol. 2020 Mar 25;11:487. doi: 10.3389/fmicb.2020.00487 (PMC7109288; doi:10.3389/fmicb.2020.00487)
Supplement: Supplementary file 2 [file Data_Sheet_2.pdf]

SUPPLEMENTARY TABLES

Table S1. Codes and numbers of nymphs and females analyzed (in parentheses) in the control population at G1 and in vancomycin and ampicillin experiments at G1 and G2. 113 samples (26 at G1 and 87 at G2) from 36 time points were analyzed. See Figure 1 for details.

	**Vancomycin experiment**			**Ampicillin experiment**			**Control**
**GENERATION 1 (G1)**	**V population**			**A population**			**C population**
0a (females 0 days)	–			–			C0a (4)
10a (females 10 days)	V10a (3)			A10a (3)			C10a (4)
30a (females 30 days)	V30a (4)			A30a (4)			C30a (4)
**TOTAL 26**	**7**			**7**			**12**
**GENERATION 2 (G2)**	**VV**	**VC**	**VF**	**AA**	**AC**	**AF**	
22n (nymphs 22 days)	VV22n (3)	VC22n (3)	VF22n (3)	AA22n (3)	AC22n (3)	AF22n (3)	–
34n (nymphs 34 days)	VV34n (3)	VC34n (3)	VF34n (3)	AA34n (3)	AC34n (3)	AF34n (3)	–
0a (females 0 days)	VV0a (3)	VC0a (3)	VF0a (3)	AA0a (3)	AC0a (3)	AF0a (3)	–
10a (females 10 days)	VV10a (3)	VC10a (3)	VF10a (3)	AA10a (3)	AC10a (3)	AF10a (3)	–
30a (females 30 days)	VV30a (3)	VC30a (3)	VF30a (3)	AA30a (3)	–	AF30a (3)	–
**TOTAL 87**	**15**	**15**	**15**	**15**	**12**	**15**	

Table S2. Number of raw (and bases), quality-host filtered and assembled reads per sample.

Sample	Raw reads	Bases	Non-host reads	Assembled reads
C0a_2	316190	108993708	217816	96154
C0a_3	357140	120859488	292567	65319
C0a_4	485254	158302567	413719	70819
C0a_5	508687	158825842	407388	99474
V10a_2	446333	152772637	302165	88167
V10a_3	439921	133643841	319324	80283
V10a_4	552277	164451167	411941	99091
A10a_1	266685	93354346	190544	63077
A10a_2	125639	48899828	94396	31335
A10a_3	226353	81180688	164697	55057
C10a_1	1098755	183053737	339215	639091
C10a_2	356703	115425605	174924	153955
C10a_3	268048	98024096	148816	104047
C10a_4	307521	109271650	174397	112324
V30a_1	154174	57308120	110207	40188
V30a_2	284150	98247884	200536	66984
V30a_3	266919	93321737	191859	63101
V30a_4	307771	106532834	237293	63309
A30a_1	646238	168670874	321402	202460
A30a_2	693581	178869790	425576	196249
A30a_3	932911	223921220	734158	140951
A30a_4	661156	183330331	423239	191076
C30a_1	301858	104281046	227071	73271
C30a_2	235685	85677516	150211	83016
C30a_3	242504	89423414	161052	78900
C30a_4	614575	161493961	423191	165041
AA34n_1	1227516	331373978	905924	258923
VV34n_1	723577	198143519	492248	133448
AA34n_2	885469	231011643	638900	205910
VV34n_2	727146	198664974	502722	116256
AA34n_3	925599	239293873	664343	225399
VV34n_3	777872	210852610	544452	126521
AC34n_1	729811	201082609	572504	146846
VC34n_1	864541	232203550	467224	150818
AC34n_2	1012401	266216636	865893	137932
VC34n_2	555188	156453837	358188	106427
AC34n_3	802543	220132837	704076	92457
VC34n_3	981971	257408559	672160	150337
AF34n_1	1101765	298066742	423928	518820
VF34n_1	655827	184932980	227499	359294
AF34n_2	757963	206391630	391329	324193
VF34n_2	893777	249808168	545875	286035
AF34n_3	1071312	281635719	558091	432343
VF34n_3	452872	123327387	225781	211139
AA0a_1	1004662	268657763	786425	189782

VV0a_1	763454	211041024	647782	105236
AA22n_1	706961	191073900	471612	162579
VV22n_1	707778	186999807	439737	108804
AA0a_2	1175408	307901437	893644	251196
VV0a_2	718955	194060789	584163	119324
AA0a_3	762021	198960672	641965	109284
VV0a_3	542812	159276589	424737	104445
AA10a_1	736520	201392993	438111	172042
VV10a_1	671137	182585693	501395	119622
AA10a_2	933429	237463762	416900	212685
VV10a_2	909804	251779144	681299	153827
AA10a_3	889493	230852643	623695	208847
VV10a_3	513528	154415186	375880	95465
AA30a_1	936724	241028709	753842	163267
VV30a_1	748253	198395067	496257	130382
AA30a_2	682582	183585192	503517	155144
VV30a_2	579067	164993538	429956	111915
AA30a_3	748546	196644607	587445	140046
VV30a_3	800969	220423536	556339	140554
AC0a_1	1828796	437690220	1411650	368537
VC0a_1	587924	158885760	490358	87433
AC0a_2	1178029	279001494	839328	301633
VC0a_2	777969	204471409	622335	141847
AA22n_2	498662	135732054	316805	145107
VV22n_2	507151	144430726	319785	89687
AC0a_3	1007407	250271874	765788	215101
VC0a_3	510998	141681271	395475	91599
AC10a_1	1174901	268458687	705012	304425
VC10a_1	594623	162448775	342048	190004
AC10a_2	1326680	300588244	710826	448999
VC10a_2	774515	193162042	433227	210386
AC10a_3	885757	201757365	491283	296968
VC10a_3	749955	200872073	455374	188207
AF0a_1	1135454	271756098	909107	208807
VC30a_1	654500	181272620	389179	191411
AF0a_2	1469263	302839692	1166917	270014
VC30a_2	712547	201091991	517507	159499
AF0a_3	1284769	310990999	1023419	229127
VC30a_3	660038	182619547	472274	153765
AF10a_1	1254111	281672905	743638	380757
VF0a_1	859789	231925997	640831	206983
AF10a_2	958390	210043325	507903	348281
VF0a_2	680235	187461040	539711	131154
AF10a_3	714986	198563681	427319	252403
VF0a_3	915519	247247988	728863	174840
AA22n_3	628048	170113513	416000	164117
VV22n_3	815821	225115669	558960	122552
AF30a_1	978990	242969741	608834	289883

VF10a_1	766817	209065090	417183	287683
AF30a_2	802930	202217256	445173	292503
VF10a_2	850381	223573028	482878	293382
AF30a_3	1026743	266986692	602240	328582
VF10a_3	536315	137777757	316604	193451
VF30a_1	713695	189615436	359483	276978
VF30a_2	852814	214438645	605646	209612
VF30a_3	785120	200464225	542496	210784
AC22n_1	953674	262679763	639337	251678
VC22n_1	714951	197509437	474786	79881
AC22n_2	798663	219876900	575396	194992
VC22n_2	765415	208552918	379404	69403
AC22n_3	652792	195203343	363685	260720
VC22n_3	869830	223130047	412801	95431
AF22n_1	1452951	423749428	672263	596637
VF22n_1	763691	210226882	373856	294418
AF22n_2	825154	234155461	365528	356228
VF22n_2	974930	185631396	452002	449195
AF22n_3	550445	151364249	231677	283466
VF22n_3	681228	181675770	283144	323516

Table S3. Relative abundance (> 0.1 % in at least one of the samples) of phyla at the different time points analyzed.

A) Vancomycin experiment

Phyla	C0a	C10a	C30a	V10a	V30a	VV22n	VV34n	VV0a	VV10a	VV30a	VC22n	VC34n	VC0a	VC10a	VC30a	VF22n	VF34n	VF0a	VF10a	VF30a
Bacteroidetes	68.14	65.49	64.41	40.59	46.11	44.28	43.20	30.16	43.54	51.62	73.87	64.56	64.45	65.76	66.61	61.11	65.79	64.07	64.91	65.23
Firmicutes	15.23	15.89	16.99	2.96	3.86	2.04	2.65	3.69	2.94	3.97	5.13	13.88	17.09	19.69	19.00	22.32	18.90	20.23	14.52	15.76
Proteobacteria	10.92	8.48	9.93	33.55	21.10	33.10	32.76	49.36	33.91	28.97	17.49	18.12	14.33	10.78	10.17	10.40	8.24	9.64	8.71	11.26
Fusobacteria	1.85	5.95	4.92	20.53	26.06	18.39	18.78	13.17	16.99	12.24	0.05	0.10	0.12	0.11	0.13	1.75	3.17	1.39	8.23	3.48
Actinobacteria	0.48	0.46	0.47	0.18	0.27	0.19	0.21	0.28	0.23	0.24	0.11	0.18	0.52	0.46	0.59	0.52	0.48	0.79	0.38	0.48
Synergistetes	0.23	0.30	0.22	0.11	0.21	0.10	0.13	0.18	0.15	0.24	0.01	0.06	0.18	0.24	0.42	0.31	0.24	0.22	0.18	0.24
Spirochaetes	0.29	0.29	0.28	0.15	0.29	0.11	0.16	0.20	0.16	0.20	0.06	0.13	0.25	0.25	0.29	0.40	0.30	0.31	0.26	0.33
Verrucomicrobia	0.16	0.18	0.16	0.12	0.12	0.05	0.07	0.07	0.07	0.11	0.07	0.07	0.10	0.08	0.09	0.12	0.14	0.16	0.23	0.16
Deferribacteres	0.15	0.14	0.11	0.14	0.31	0.14	0.29	0.41	0.21	0.43	0.00	0.01	0.00	0.01	0.01	0.03	0.05	0.03	0.03	0.04
Planctomycetes	0.11	0.14	0.11	0.09	0.09	0.05	0.09	0.09	0.09	0.11	0.02	0.03	0.06	0.06	0.07	0.14	0.10	0.11	0.09	0.10
Cyanobacteria	0.26	0.10	0.19	0.14	0.18	0.06	0.08	0.30	0.18	0.12	0.04	0.06	0.23	0.06	0.14	0.08	0.08	0.22	0.12	0.13
Candidatus Saccharibacteria	0.00	0.00	0.00	0.00	0.00	0.00	0.00	0.00	0.00	0.00	0.00	0.00	0.00	0.00	0.00	0.00	0.06	0.03	0.01	0.18
Others	2.18	2.58	2.21	1.44	1.40	1.49	1.58	2.09	1.53	1.75	3.15	2.80	2.67	2.50	2.48	2.82	2.45	2.80	2.33	2.61

A) Ampicillin experiment.

Phyla	C0a	C10a	C30a	A10a	A30a	AA22n	AA34n	AA0a	AA10a	AA30a	AC22n	AC34n	AC0a	AC10a	AF22n	AF34n	AF0a	AF10a	AF30a
Bacteroidetes	68.14	65.49	64.41	77.21	67.08	71.64	64.15	53.43	69.87	66.79	71.59	68.32	58.11	70.03	56.45	63.23	65.10	61.46	65.92
Firmicutes	15.23	15.89	16.99	2.14	9.41	6.90	12.14	20.42	7.03	11.24	15.76	17.57	26.34	17.21	23.20	20.14	16.89	14.25	14.02
Proteobacteria	10.92	8.48	9.93	10.85	10.61	10.46	12.49	15.11	7.19	10.06	6.66	9.98	10.86	9.17	9.81	8.68	11.14	8.69	11.33
Fusobacteria	1.85	5.95	4.92	7.68	9.43	7.07	4.95	6.87	12.34	7.96	2.68	0.11	0.15	0.11	6.48	3.73	1.99	12.07	4.79
Actinobacteria	0.48	0.46	0.47	0.10	0.33	0.29	1.07	0.66	0.20	0.65	0.32	0.44	0.69	0.30	0.47	0.46	0.73	0.39	0.37
Synergistetes	0.23	0.30	0.22	0.02	0.10	0.11	0.23	0.13	0.09	0.08	0.12	0.12	0.16	0.15	0.28	0.32	0.24	0.16	0.22
Spirochaetes	0.29	0.29	0.28	0.06	0.19	0.25	0.55	0.28	0.18	0.18	0.21	0.32	0.38	0.27	0.39	0.32	0.27	0.23	0.28
Verrucomicrobia	0.16	0.18	0.16	0.06	0.08	0.10	0.16	0.10	0.09	0.10	0.08	0.10	0.09	0.08	0.15	0.22	0.35	0.24	0.19
Deferribacteres	0.15	0.14	0.11	0.00	0.06	0.01	0.02	0.01	0.01	0.01	0.03	0.01	0.01	0.01	0.06	0.07	0.07	0.07	0.05
Planctomycetes	0.11	0.14	0.11	0.04	0.07	0.15	0.35	0.12	0.08	0.11	0.06	0.07	0.08	0.09	0.10	0.11	0.10	0.08	0.09
Cyanobacteria	0.26	0.10	0.19	0.07	0.13	0.11	0.19	0.31	0.07	0.19	0.09	0.31	0.19	0.09	0.10	0.07	0.25	0.11	0.11
Lentisphaerae	0.03	0.04	0.03	0.01	0.03	0.05	0.13	0.03	0.02	0.02	0.01	0.03	0.04	0.03	0.04	0.04	0.04	0.03	0.04
Others	2.15	2.54	2.18	1.76	2.48	2.86	3.57	2.53	2.83	2.61	2.39	2.62	2.90	2.46	2.47	2.61	2.83	2.22	2.59

Table S4 (.xls). Relative abundance (> 0.1% in at least one of the samples) of families of the different time points analyzed. A) Vancomycin experiment. B) Ampicillin experiment.

See separate Excel file Table S4

Table S5. Relative abundance (> 0.1%) of the families (or higher taxonomic level) present in C10a, C30a, VF10a, VF30a, AF10a and AF30a samples that form the core of adults gut microbiota (see text for details).

Phylum	Family	% of abundance	SD
Bacteroidetes	Porphyromonadaceae	28.00	1.52
	Bacteroidaceae	12.98	1.99
	Rikenellaceae	5.16	0.90
	Prevotellaceae	4.78	0.34
	Bacteroidales_uc	4.12	0.29
	Bacteroidetes_uc	2.27	0.17
	Flavobacteriaceae	1.58	0.09
	Odoribacteraceae	1.18	0.33
	Sphingobacteriaceae	1.00	0.05
	Bacteroidia_uc	0.97	0.05
	Marinilabiliaceae	0.46	0.04
	Chitinophagaceae	0.43	0.02
	Prolixibacteraceae	0.37	0.02
	Cytophagaceae	0.29	0.01
	Cyclobacteriaceae	0.16	0.01
	Flammeovirgaceae	0.13	0.01
	Hymenobacteraceae	0.12	0.01
Firmicutes	Ruminococcaceae	3.05	0.30
	Clostridiaceae	2.54	0.22
	Clostridiales_uc	2.41	0.23
	Lachnospiraceae	2.31	0.47
	Firmicutes_uc	0.74	0.07
	Eubacteriaceae	0.44	0.04
	Peptococcaceae	0.43	0.03
	Enterococcaceae	0.37	0.12
	Erysipelotrichaceae	0.36	0.07
	Paenibacillaceae	0.30	0.03
	Bacillaceae	0.29	0.02
	Sporomusaceae	0.25	0.01
	Peptostreptococcaceae	0.19	0.02
	Oscillospiraceae	0.15	0.02
Proteobacteria	Desulfovibrionaceae	4.10	0.67
	Deltaproteobacteria_uc	0.74	0.12
	Enterobacteriaceae	0.42	0.25
	Desulfobacteraceae	0.23	0.02
	Oxalobacteraceae	0.13	0.02
	Desulfomicrobiaceae	0.13	0.02
Fusobacteria	Fusobacteriaceae	6.51	3.11
Actinobacteria	Eggerthellaceae	0.13	0.02
Spirochaetes	Spirochaetaceae	0.22	0.03
Synergistetes	Synergistaceae	0.20	0.05

Table S6. Relative abundance of main functional roles at the different time point analyzed. Main roles were ordered in functions of relative abundance of C10a values.

A) Vancomycin experiment.

MAIN ROLE	C0a	C10a	C30a	V10a	V30a	VV22n	VV34n	VV0a	VV10a	VV30a	VC22n	VC34n	VC0a	VC10a	VC30a	VF22n	VF34n	VF0a	VF10a	VF30a
**Transport and binding proteins**	16.41	18.64	18.45	20.51	20.60	22.38	21.77	16.20	20.87	21.61	24.79	22.44	20.79	22.15	19.92	20.17	19.49	17.10	19.27	18.13
**Energy metabolism**	9.22	11.11	10.31	10.85	9.89	11.41	11.23	9.61	10.65	10.55	9.54	10.13	10.03	10.50	10.57	10.88	10.93	10.33	11.07	11.14
**Protein synthesis**	8.20	9.90	9.00	7.40	7.16	6.89	7.34	6.99	7.19	7.02	6.16	7.16	8.20	7.88	8.13	8.54	9.14	9.34	9.52	9.63
**DNA metabolism**	7.36	8.04	7.77	6.47	6.62	6.59	6.28	5.40	6.30	6.48	8.00	7.92	7.40	7.94	8.08	7.94	8.08	7.45	7.95	8.02
**Cellular processes**	6.44	7.27	7.14	8.13	7.27	8.41	8.04	6.62	7.54	8.39	7.97	7.95	7.21	7.93	7.87	7.74	7.66	6.74	7.43	7.57
**Protein fate**	6.23	7.25	6.97	7.37	7.02	8.11	8.12	8.27	7.39	7.48	7.92	7.54	5.99	6.84	6.64	6.70	7.08	6.03	7.22	7.27
**Cell envelope**	5.19	5.49	5.26	4.79	4.88	4.89	4.92	4.04	4.64	5.00	5.66	5.48	5.20	5.44	5.56	5.31	5.64	5.34	5.65	5.46
**Biosynthesis of cofactors, prosthetic groups, and carriers**	4.64	5.33	5.20	5.61	5.05	5.82	5.76	4.56	5.39	5.48	4.94	5.07	4.65	4.97	4.91	5.16	5.23	5.09	5.41	5.50
**Amino acid biosynthesis**	3.75	4.74	4.23	3.69	3.47	3.68	3.87	3.75	3.60	3.76	3.42	3.63	3.81	4.18	4.08	4.51	4.58	4.22	4.58	4.54
**Regulatory functions**	3.39	3.88	3.86	4.25	3.96	4.13	4.35	3.79	4.01	4.19	3.16	3.82	4.00	4.06	4.17	4.60	4.04	3.71	3.67	3.86
**Purines, pyrimidines, nucleosides, and nucleotides**	2.83	3.69	3.37	2.75	2.66	2.67	2.69	2.55	2.55	2.71	2.43	2.77	2.62	3.10	3.05	3.41	3.45	3.27	3.51	3.53
**Unknown function**	2.95	3.53	3.35	3.14	2.81	3.20	3.14	2.50	4.47	2.96	2.95	3.18	2.99	3.21	3.32	3.46	3.48	3.18	3.45	3.38
**Signal transduction**	2.58	2.90	2.61	2.89	2.62	3.34	3.04	2.23	2.68	3.29	5.95	4.92	3.28	3.95	3.37	3.62	3.04	2.64	2.53	2.60
**Central intermediary metabolism**	1.92	2.28	2.19	2.40	2.39	2.42	2.50	2.03	2.43	2.60	1.87	2.08	2.00	2.16	2.14	2.27	2.18	2.00	2.15	2.25
**Transcription**	1.75	2.21	1.96	1.67	1.59	1.54	1.61	1.51	1.56	1.66	1.45	1.60	1.60	1.78	1.73	1.90	2.01	1.85	2.04	2.07
**Mobile and extrachromosomal element functions**	15.44	1.73	6.34	5.69	9.93	2.25	3.07	17.94	6.58	4.56	1.81	2.33	8.44	2.03	4.63	1.60	2.07	9.98	2.64	3.12
**Fatty acid and phospholipid metabolism**	1.05	1.30	1.19	1.54	1.32	1.47	1.44	1.24	1.42	1.47	1.23	1.22	1.10	1.18	1.14	1.40	1.17	1.06	1.17	1.16
**Hypothetical proteins**	0.62	0.70	0.78	0.82	0.75	0.80	0.79	0.73	0.73	0.77	0.75	0.75	0.68	0.70	0.68	0.77	0.71	0.64	0.72	0.75
**Unclassified**	0.01	0.02	0.01	0.01	0.01	0.01	0.02	0.02	0.01	0.01	0.00	0.01	0.02	0.01	0.01	0.02	0.01	0.01	0.01	0.01

B) Ampicillin experiment.

MAIN ROLE	C0a	C10a	C30a	A10a	A30a	AA22n	AA34n	AA0a	AA10a	AA30a	AC22n	AC34n	AC0a	AC10a	AF22n	AF34n	AF0a	AF10a	AF30a
**Transport and binding proteins**	16.41	18.64	18.45	23.11	20.36	23.49	20.86	19.12	23.38	19.54	19.99	14.83	17.92	19.73	19.35	18.76	16.88	18.42	18.81
**Energy metabolism**	9.22	11.11	10.31	10.14	10.61	10.14	10.62	10.71	9.89	10.49	10.57	10.75	10.75	10.85	10.60	10.69	10.98	10.93	10.82
**ynthesis**	8.20	9.90	9.00	7.13	9.01	7.65	7.89	8.25	7.54	9.18	9.16	9.10	9.50	9.42	8.97	9.35	9.30	9.99	9.49
**DNA metabolism**	7.36	8.04	7.77	6.89	7.94	7.63	7.05	8.03	7.94	7.89	8.10	8.14	7.93	8.28	8.24	8.39	7.86	8.13	7.96
**Cellular processes**	6.44	7.27	7.14	7.32	7.43	7.44	7.31	6.73	7.50	6.76	7.63	5.96	7.25	7.60	7.87	7.82	6.78	7.36	7.44
**Protein fate**	6.23	7.25	6.97	6.52	7.51	6.61	6.21	6.16	7.23	6.52	6.90	5.74	6.63	7.43	7.10	7.17	6.32	7.32	7.14
**Cell envelope**	5.19	5.49	5.26	4.97	5.40	5.50	4.69	4.77	5.53	5.08	5.79	5.96	5.49	5.72	5.41	5.61	5.24	5.51	5.52
**Biosynthesis of cofactors, prosthetic groups, and carriers**	4.64	5.33	5.20	5.07	5.54	4.89	4.90	5.05	5.11	5.30	5.22	4.63	5.03	5.48	5.15	5.29	4.94	5.54	5.55
**Amino acid biosynthesis**	3.75	4.74	4.23	3.88	4.24	4.35	4.22	4.11	3.77	4.21	4.31	3.67	4.48	4.59	4.28	4.55	4.42	4.57	4.50
**Regulatory functions**	3.39	3.88	3.86	3.13	3.52	3.85	4.25	4.00	3.22	3.40	3.69	2.86	4.05	3.49	4.55	4.21	3.31	3.51	3.56
**Purines, pyrimidines, nucleosides, and nucleotides**	2.83	3.69	3.37	2.56	3.53	3.04	3.14	3.19	2.91	3.38	3.36	2.77	3.53	3.55	3.48	3.51	3.47	3.65	3.46
**Unknown function**	2.95	3.53	3.35	2.85	3.26	2.99	3.11	3.21	3.08	3.21	3.40	4.04	3.37	3.44	3.46	3.44	3.09	3.38	3.44
**Signal transduction**	2.58	2.90	2.61	3.05	2.78	3.41	2.86	2.98	4.42	2.59	2.78	2.04	2.98	2.81	3.28	3.04	2.47	2.40	2.45
**Central intermediary metabolism**	1.92	2.28	2.19	2.07	2.23	2.16	2.22	2.19	2.08	2.14	2.06	1.65	2.18	2.13	2.24	2.24	2.02	2.24	2.21
**Transcription**	1.75	2.21	1.96	1.58	1.93	1.68	1.75	1.83	1.69	1.86	1.87	1.72	2.06	2.01	2.00	2.05	2.03	2.14	2.02
**Mobile and extrachromosomal element functions**	15.44	1.73	6.34	7.89	2.77	3.26	7.02	7.80	2.73	6.56	3.25	14.60	4.94	1.58	2.02	1.87	8.99	2.89	3.65
**Fatty acid and phospholipid metabolism**	1.05	1.30	1.19	1.04	1.19	1.21	1.18	1.13	1.19	1.14	1.20	1.01	1.18	1.21	1.22	1.22	1.25	1.28	1.19
**Hypothetical proteins**	0.62	0.70	0.78	0.82	0.76	0.69	0.70	0.72	0.78	0.72	0.71	0.54	0.73	0.67	0.78	0.76	0.61	0.74	0.78
**Unclassified**	0.01	0.02	0.01	0	0.01	0.01	0.02	0.03	0.01	0.01	0.01	0.00	0.02	0.01	0.01	0.01	0.02	0.01	0.01

Table S7 (.xls) Relative abundance of the subroles, belonging to each main role, found at different time point analyzed. A) Vancomycin experiment. B) Ampicillin experiment.

See separate Excel file Table S7

Table S8 (.xls) Number of reads of the genes found in all samples analyzed (113 samples).

See separate Excel file Table S8

Table S9 (.xls) Relative abundance of main roles and sub roles present in C10a, C30a, VF10a, VF30a, AF10a and AF30a samples that form the core of adults gut microbiota.

See separate Excel file Table S9

Table S10. Antibiotic resistance genes detected in the control and antibiotic treated samples.

Time sampling	ARGs	Resistance profile	Phylum
C0a, C10a, C30a	*acrB*	aminoglycoside, glycylcycline, beta_lactam, macrolide, acriflavin	Proteobacteria, Verrucomicrobia, Deferribacteres
	bacA	bacitracin	Bacteroidetes, Firmicutes, Proteobacteria
	mexB	tigecycline, aminoglycoside, fluoroquinolone, beta_lactam, tetracycline	Proteobacteria, Verrucomicrobia
	tetM	tetracycline	Bacteria_uc, Firmicutes
**Vancomycin treatment**			
V10a, V30a	*acrB*	aminoglycoside, glycylcycline, beta_lactam, macrolide, acriflavin	Proteobacteria
	bacA	bacitracin	Bacteroidetes, Fusobacteria, Proteobacteria, Firmicutes
	mexB	tigecycline, aminoglycoside, fluoroquinolone, beta_lactam, tetracycline	Proteobacteria
VV22n, VV34n	*acrB*	aminoglycoside, glycylcycline, beta_lactam, macrolide, acriflavin	Proteobacteria
	bacA	bacitracin	Bacteroidetes, Fusobacteria, Proteobacteria
	macB	macrolide	Proteobacteria
	mdtK	enoxacin, norfloxacin	Proteobacteria
	mexB	tigecycline, aminoglycoside, fluoroquinolone, beta_lactam, tetracycline	Proteobacteria
	rosA	fosmidomycin	Proteobacteria
	smeE	fluoroquinolone	Proteobacteria
VV0a, VV10a, VV30a	*acrB*	aminoglycoside, glycylcycline, beta_lactam, macrolide, acriflavin	Proteobacteria, Verrucomicrobia
	bacA	bacitracin	Bacteroidetes, Fusobacteria, Proteobacteria
	mexB	tigecycline, aminoglycoside, fluoroquinolone, beta_lactam, tetracycline	Proteobacteria, Bacteria
	rosA	fosmidomycin	Bacteroidetes, Proteobacteria
	smeE	fluoroquinolone	Proteobacteria
**Ampicillin treatment**			
A10a, A30a	*bacA*	bacitracin	Bacteroidetes, Firmicutes, Fusobacteria, Proteobacteria
AA22n, AA34n	*acrB*	aminoglycoside, glycylcycline, beta_lactam, macrolide, acriflavin	Proteobacteria
	bacA	bacitracin	Actinobacteria, Bacteroidetes, Fusobacteria, Proteobacteria, Firmicutes
	ksgA	kasugamycin	Proteobacteria
	mexB	tigecycline, aminoglycoside, fluoroquinolone, beta_lactam, tetracycline	Proteobacteria
AA0a, AA10a, AA30a	*acrB*	aminoglycoside, glycylcycline, beta_lactam, macrolide, acriflavin	Bacteria_uc, Deferribacteres, Proteobacteria, Verrucomicrobia
	bacA	bacitracin	Bacteria_uc, Bacteroidetes, Firmicutes, Fusobacteria, Proteobacteria
	macB	macrolide	Proteobacteria
	mexB	tigecycline, aminoglycoside, fluoroquinolone, beta_lactam, tetracycline	Proteobacteria
	rosB	fosmidomycin	Bacteria_uc, Proteobacteria
	tetM	tetracycline	Bacteria_uc

It shows the ARGs that were present in at least two of the biological replicas in at least two of each grouped sampling time.
